# An observational cohort study to investigate the impact of dolutegravir in pregnancy and its obesogenic effects on the metabolic health of women living with HIV and their children: Study protocol

**DOI:** 10.1371/journal.pone.0307296

**Published:** 2024-08-19

**Authors:** Elaine J. Abrams, Jennifer Jao, Hlengiwe P. Madlala, Allison Zerbe, Patrick Catalano, Mariana Gerschenson, Julia H. Goedecke, Yolanda Gomba, Jami Josefson, Irwin J. Kurland, Justine Legbedze, Grace A. McComsey, Sandisiwe Matyesini, Elton Mukonda, Daniel Robinson, Landon Myer

**Affiliations:** 1 ICAP at Columbia University, Mailman School of Public Health, Columbia University, New York, NY, United States of America; 2 Department of Epidemiology, Mailman School of Public Health, Columbia University, New York, NY, United States of America; 3 Department of Pediatrics, Vagelos College of Physicians & Surgeons, Columbia University, New York, NY, United States of America; 4 Department of Pediatrics, Northwestern University Feinberg School of Medicine, Chicago, IL, United States of America; 5 Division of Epidemiology and Biostatistics, School of Public Health, University of Cape Town, Western Cape, Cape Town, South Africa; 6 Maternal Infant Research Institute, Obstetrics and Gynecology Research, Gerald J. and Dorothy R. Friedman School of Nutrition Science and Policy, Tufts University School of Medicine, Tufts University, Boston, MA, United States of America; 7 Department of Cell and Molecular Biology, John A. Burns School of Medicine, University of Hawaii at Manoa, Honolulu, HI, United States of America; 8 Biomedical Research and Innovation Platform, South African Medical Research Council, Cape Town, South Africa; 9 Health through Physical Activity, Lifestyle and Sport Research Centre (HPALS), Division of Physiological Sciences, Department of Human Biology, University of Cape Town, Cape Town, South Africa; 10 Department of Medicine, Stable Isotope and Metabolomics Core Facility, Albert Einstein College of Medicine, Bronx, NY, United States of America; 11 University Hospitals Health System, Cleveland, OH, United States of America; 12 Department of Medicine and Pediatrics of Case Western Reserve University, Cleveland, OH, United States of America; Public Library of Science, UNITED KINGDOM OF GREAT BRITAIN AND NORTHERN IRELAND

## Abstract

**Introduction:**

Dolutegravir (DTG)-based antiretroviral therapy is the World Health Organization’s preferred first-line regimen for all persons with HIV, including pregnant women. While DTG has been implicated as an obesogen associated with greater weight gain compared to other antiretrovirals, there is a paucity of data in pregnant women and their children. The Obesogenic oRigins of maternal and Child metabolic health Involving Dolutegravir (ORCHID) study is investigating associations between DTG, weight gain, and metabolic outcomes in the context of HIV.

**Materials & methods:**

ORCHID is a prospective observational study taking place in Cape Town, South Africa (NCT 04991402). A total of 1920 pregnant women with and without HIV infection are being followed from ≤18 weeks gestational age to 24 months postpartum with their children. Participants attend eleven study visits: 3 antenatal, delivery, and 7 postnatal visits. Several embedded sub-studies address specific scientific aims. Primary outcome measurements in mothers include anthropometry, blood pressure, body composition, dysglycemia, insulin resistance (IR), and dyslipidemia. Other maternal measures include demographics, resting energy expenditure, viral load, physical activity, dietary intake, hepatic steatosis, and repository specimens. Sub-study measurements include markers of adipose inflammation, gut integrity, and satiety/hunger, subcutaneous adipose tissue morphology and mitochondrial function, and metabolomics. Primary outcome measurements in children include anthropometry, adipose tissue mass, dysglycemia, IR, and dyslipidemia. Other variables include fetal growth, birth outcomes, medical/breastfeeding history, caloric intake, neurodevelopment, and repository specimens. Sub-study measurements include metabolites/lipid subspecies in umbilical cord blood, as well as breast milk composition and DTG exposure.

**Discussion:**

ORCHID will play a pivotal role in defining obesogenic mechanisms and clinical consequences of DTG use in pregnancy in women with HIV and their children. It will provide insights into metabolic disease risk reduction in the context of HIV/DTG, identify intervention targets, and inform public health approaches to diminish chronic metabolic co-morbidities for women and children.

## Introduction

The introduction and scale-up of antiretroviral treatment (ART) for HIV infection has led to stunning improvements in health outcomes among people with HIV (PWH), resulting in dramatic reductions in HIV-attributed mortality and morbidity globally [1]. Each generation of antiretrovirals agents, however, has been saddled with short and long-term toxicities, often limiting treatment efficacy and adherence to drug regimens [2]. In 2019, the World Health Organization (WHO) updated antiretroviral treatment (ART) guidelines to recommend dolutegravir (DTG), an Integrase Strand Transfer Inhibitor (INSTI), with lamivudine (3TC) and tenofovir (TDF) as the preferred first-line regimen for all individuals living with HIV, including women of childbearing age, due to the high potency, excellent tolerability, and low toxicity of the once-daily fixed-dose combination regimen (TDF+3TC+DTG [TLD]) [3, 4].

Weight gain after ART initiation is common among individuals with HIV infection, and adverse metabolic and body composition changes have been associated with specific antiretroviral (ARV) agents as well as ARV classes and regimens [5]. Several observational studies reported a potential association between INSTI use and excessive weight gain compared with alternative regimens [6–11]. The ADVANCE trial, a phase 3, open-label, randomized trial comparing two DTG-based regimens to the non-nucleoside reverse transcriptase inhibitor efavirenz (EFV)-based ART in individuals initiating treatment in South Africa found significantly more weight gain and a higher rate of incident obesity with the DTG-containing regimens among women [12]. Similarly, the NAMSAL trial, another study comparing DTG- to EFV-based ART conducted in Cameroon, found a greater mean increase in body weight and emergent obesity among participants in the DTG group [13]. These findings are particularly alarming in the context of escalating rates of overweight and obesity globally including among PWH and in low- and middle-income countries [13, 14]. In 2020, it was estimated that almost 1 billion people were living with obesity (body mass index (BMI) ≥30kg/m^2^) and the prevalence of obesity is predicted to increase to 24% of the world population by 2035 [14]. In South Africa, over half of the adult population are overweight or living with obesity [15].

Obesity during pregnancy has particular implications for health. Pregnancy is a critical period in the life course that influences both long-term maternal and child health outcomes [16]. Excess gestational weight gain (GWG) in pregnancy is associated with preeclampsia, gestational diabetes mellitus (GDM), and long-term adiposity and obesity [17–24]. Women with excessive GWG experience a two-fold risk of postpartum weight retention [25] and a significantly higher risk (47%) of developing diabetes over 20 years postpartum compared to those with appropriate GWG [26]. Furthermore, women with excessive early GWG are more likely to have infants with higher birth weight, adiposity, and insulin resistance (IR) [27–31]. The observational Tsepamo study in Botswana and the International Maternal Pediatric Adolescent AIDS Clinical Trials Network (IMPAACT) Study 2010 phase 3 safety and efficacy study of DTG versus EFV-containing ART among women living with HIV (WHIV) initiating ART during pregnancy both reported higher GWG among WHIV receiving DTG-based ART [32, 33]. Despite these findings, there is a paucity of data on DTG and its obesogenic effects, including the disruption of metabolic control and promotion of adipogenesis and lipid accumulation, in pregnant WHIV and their children. With approximately 1.3 million WHIV becoming pregnant annually, and expectations that over 90% will be treated with TLD, the impact on maternal and child metabolic health outcomes may be profound [1].

### The Obesogenic oRigins of maternal and Child metabolic health Involving Dolutegravir study

To address this knowledge gap, the Obesogenic oRigins of maternal and Child metabolic health Involving Dolutegravir (ORCHID) study is investigating the impact of DTG in pregnancy and its obesogenic effects on the metabolic health of WHIV and their children, compared to women without HIV and their children. ORCHID is a prospective observational cohort study, with an embedded set of nested sub-studies, of WHIV on DTG-based ART and a comparison group of women without HIV infection, enrolled during pregnancy, and followed with their children through two years postpartum. This study will provide key insights on the impact of HIV and DTG on metabolic health outcomes of mothers and their children and inform public health approaches to optimize HIV treatment and reduce chronic co-morbidities over the life course of WHIV and their children.

## Materials and methods

### ORCHID study aims

Based in Cape Town, South Africa, the ORCHID study is investigating how HIV and/or DTG use impacts longitudinal changes in weight, lean body mass and adipose tissue mass in pregnancy measured using air displacement plethysmography (ADP) in a population where both HIV and obesity in pregnancy are prevalent (Aim 1). Additionally, the protocol is investigating pathways linking HIV/DTG exposure to excess GWG and adipose accrual by evaluating a) the balance between caloric intake and resting energy expenditure (REE) (Aim 1a); b) markers of systemic and adipose inflammation, gut integrity, and satiety/hunger (Aim 1b); and c) subcutaneous adipose tissue (SAT) morphology and mitochondrial function (Aim 1c). The study hypothesizes that HIV/DTG use in pregnancy is (i) associated with excess GWG and accrual of adipose tissue mass, and (ii) that these associations are mediated by increases in caloric intake and satiety/hunger markers as well as lower energy expenditure compared with controls. In addition, we hypothesize that DTG use is associated with changes in SAT including adipocyte hypertrophy, hypoxia, and increased fibrosis and inflammation as well as decreased mitochondrial respiration.

The ORCHID study is also examining how HIV/DTG use in pregnancy and postpartum affects maternal metabolic health postpartum (postpartum weight retention, adiposity, dysglycemia, insulin resistance, and dyslipidemia) (Aim 2 and 2a). Further, the protocol is exploring whether a signature cluster of metabolites and lipid subspecies in pregnancy are associated with metabolic health postpartum in WHIV receiving DTG (Aim 2b). Last, the impacts of HIV/DTG exposure *in utero* on neonatal and child metabolic health through two years of life are being investigated (Aim 3). This includes specifically evaluating the association between HIV/DTG exposure and neonatal weight, adiposity, insulin resistance, and dyslipidemia (Aim 3a), signature clusters of metabolites, lipid subspecies, and eicosanoids (Aim 3b) as well as breastmilk composition (Aim 3c). We hypothesize that DTG use during pregnancy and postpartum will be associated with adverse maternal metabolic health postpartum, as well as adverse neonatal and child metabolic health, and that these associations will be mediated by excess GWG, accrual of adipose tissue mass in pregnancy and a signature cluster of metabolites and lipid subspecies and eicosanoids.

### Study design

**Fig 1:**
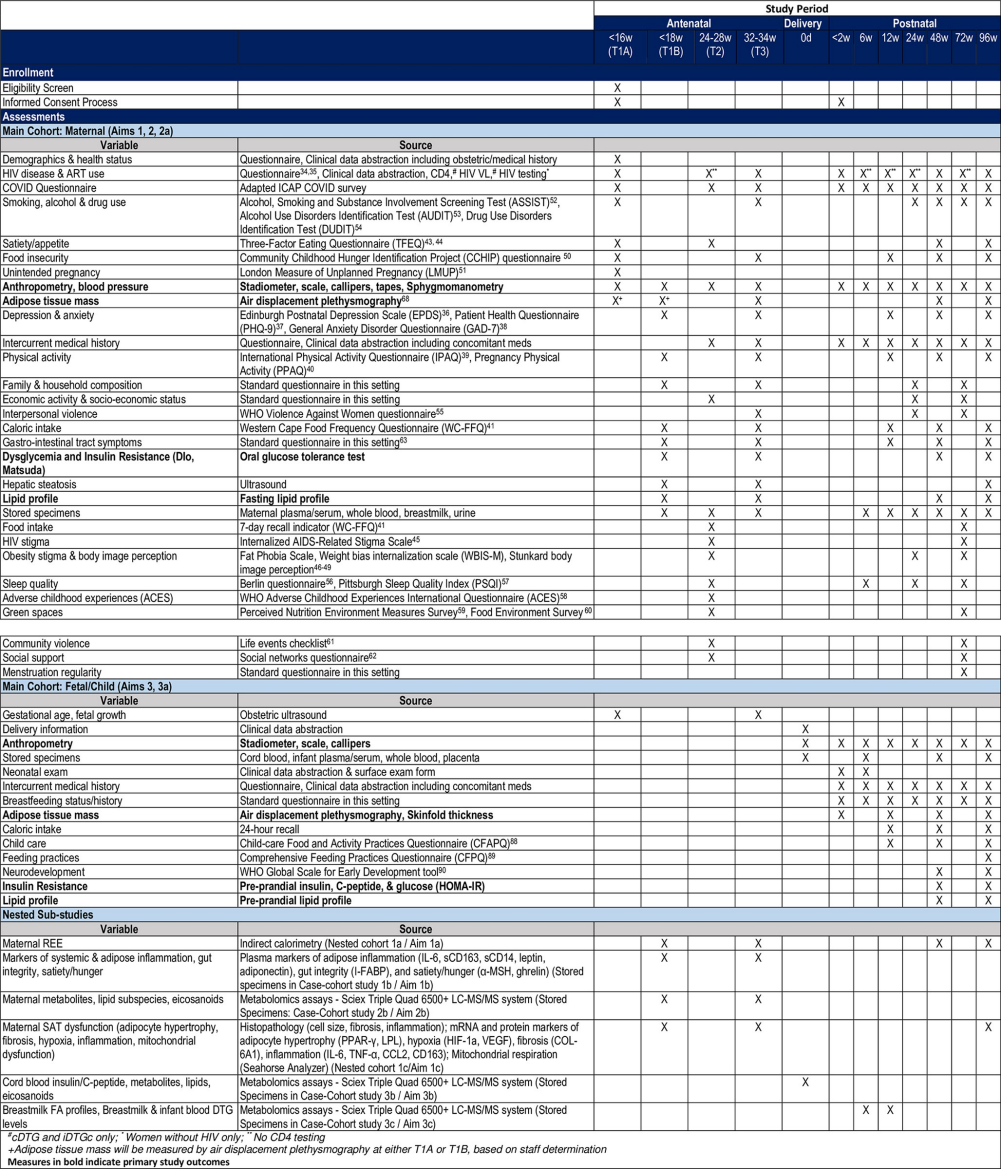
Obesogenic oRigins of maternal and Child metabolic health Involving Dolutegravir (ORCHID) study schedule of events for main cohort and nested sub-studies.

**Fig 2:**
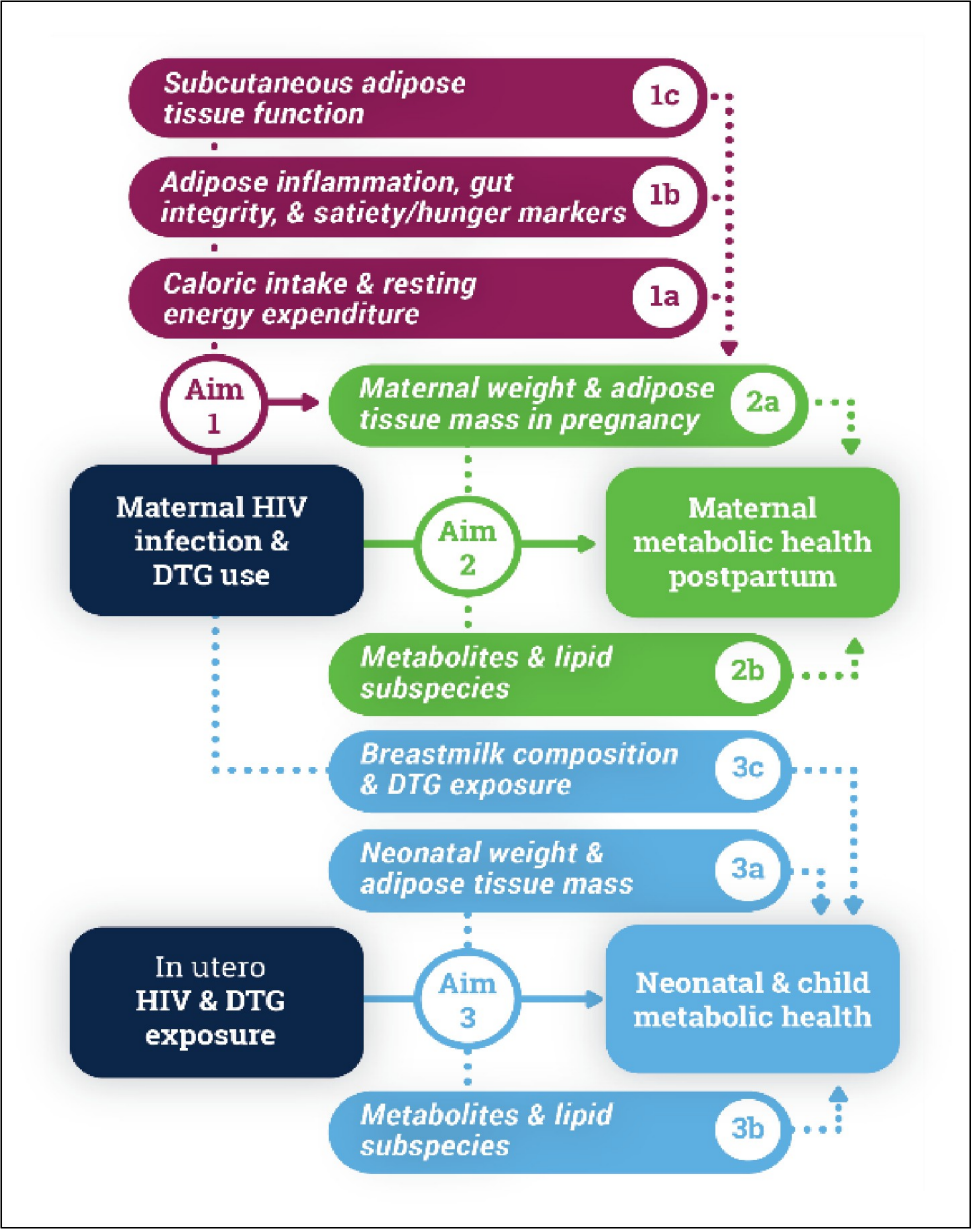
Obesogenic oRigins of maternal and Child metabolic health Involving Dolutegravir (ORCHID) study aims and sub-aims.

ORCHID is a prospective cohort study (NCT 04991402) following cisgender pregnant women with and without HIV infection from ≤18 weeks gestational age (GA) to 24 months postpartum along with their children (the Main Cohort, MC) through eleven study visits, including 3 antenatal visits (≤18, 24–28 and 32–36 weeks), sample collection at delivery, and 7 postnatal visits (<2 and 6 weeks, 3, 6, 12, 18 and 24 months). The schedule of events across these study visits is shown in Fig 1. A series of sub-studies, including smaller nested cohorts and case-cohort designs, within the MC are being used to address specific sub-aims depicted in Fig 2 and Table 1.

**Table 1:** Obesogenic oRigins of maternal and Child metabolic health Involving Dolutegravir (ORCHID) summary of study designs, comparison groups and key measures for analyses to address specific aims and sub-aims.

*Aim & Study* *(population)*		*Design and Comparisons*	*Key measures for analysis*	*Sample size for analysis*
**1**	**Main Cohort** (*mothers*)	Prospective cohort study of all eligible pregnant women, comparing (i) HIV+ vs HIV- and (ii) HIV+ continuing DTG (cDTG) vs initiating DTG (iDTG) in pregnancy	Longitudinal changes in weight, adipose tissue mass (ADP)	n = 1920
**1a**	**Nested cohort 1a** (*mothers*)	Prospective cohort study sampled from Main Cohort, comparing (i) HIV+ vs HIV- and (ii) cDTG vs iDTG	Maternal REE & Caloric intake	n = 1240
**1b**	**Case-cohort study 1b** (*mothers*)	Case-cohort sampled from within nested cohort 1a 2 case definitions: women in the top deciles of the cohort in (i) adipose accrual by ADP between T1 and T3, & (ii) GWG in pregnancy Comparator subcohort: random sample of all women in MC Comparisons by HIV+ vs HIV-; cDTG vs iDTG; and case vs comparator subcohort	Markers of systemic & adipose inflammation, gut integrity, satiety/hunger	n = 840
**2**	**Main Cohort** (*mothers*)	Prospective cohort study of all eligible pregnant women, comparing (i) HIV+ vs HIV- and (ii) cDTG vs iDTG	*Metabolic health PP*: PP weight retention; adiposity (ADP); Dysglycemia (OGTT); IR (DIo, Mari, Matsuda); Dyslipidemia	n = 1200
**2a**	**Main Cohort** (*mothers*)	Prospective cohort study of all eligible pregnant women, comparing (i) HIV+ vs HIV- and (ii) cDTG vs iDTG	Longitudinal changes in weight, adipose tissue mass (ADP), Metabolic health PP (see above)	n = 1200
**2b**	**Case-cohort study 2b** (*mothers*)	Case-cohort sampled from within MC 2 case definitions: women with (i) poorest decile of DIo values at 24m PP, & (ii) women in top decile of PP weight retention in cohort at 24m postpartum Comparator subcohort: random ***sample*** of all women in MC Comparisons by HIV+ vs HIV-; cDTG vs iDTG; and case vs comparator subcohort	Maternal metabolites, lipid subspecies, eicosanoids	n = 960
**3**	**Main Cohort** (*infants/children*)	Prospective cohort study of all eligible children, comparing (i) HIV-exposed vs HIV-unexposed and (ii) born to HIV+ mothers cDTG vs iDTG	*Neonatal metabolic health*: Weight; Adipose tissue mass (ADP, skinfold thickness); IR (Cord blood glucose, insulin, C-peptide); *Child metabolic health*: Weight; Adipose tissue mass (skinfold thickness); IR (HOMA-IR); Dyslipidemia	n = 1200
**3a**	**Main Cohort** (*infants/children*)	Prospective cohort study of all eligible children, comparing (i) HIV-exposed vs HIV-unexposed and (ii) born to HIV+ mothers cDTG vs iDTG	Neonatal adipose tissue mass (ADP) Neonatal metabolic health, child metabolic health (above)	n = 1200
**3b**	**Case-cohort study 3b** (*infants/children*)	Case-cohort sampled from children with cord blood specimens within MC 3 case definitions: top deciles of: (i) neonatal adipose tissue mass from ADP; (ii) adipose tissue mass from skinfold thicknesses at 24m & (iii) LDL at 24m Comparator subcohort: random sample of all children in MC Comparisons by HIV+ vs HIV-; cDTG vs iDTG; and case vs comparator subcohort	Cord blood insulin/C-peptide, metabolites, lipid subspecies, eicosanoids	n = 750
**3c**	**Case-cohort study 3c** (*infants/children*)	Case-cohort sampled from children in Case-cohort study 3b Case definitions as for Case-cohort study 3b Comparator subcohort: random sample of all children in MC Comparisons by HIV+ vs HIV-; cDTG vs iDTG; and case vs comparator subcohort	Breastmilk fatty acid profiles, breastmilk & infant blood DTG levels	n = 250

HIV+: Women living with HIV; HIV-: women without HIV; DTG: dolutegravir

### Study setting

Pregnant women were recruited at antenatal care facilities located in the Klipfontein-Mitchell’s Plain subdistrict in Cape Town. These community-based facilities provide comprehensive primary care, including maternal and child health services with integrated HIV/ART during pregnancy, for residents of Gugulethu and Mitchells Plain, two of the largest former township communities in Cape Town. Vertical HIV transmission rates are estimated at 2–3% through 18 months of age. Breastfeeding is widespread (>80%) regardless of maternal HIV with most women weaning around 6 months postpartum.

### Eligibility criteria

Participants are pregnant women ≤18 weeks GA at enrollment. Other inclusion criteria included confirmed HIV infection based on medical record review and/or HIV antibody testing during antenatal care (for WHIV only), confirmed HIV status by HIV antibody testing during antenatal care (HIV-seronegative participants only), and no stated intention to relocate outside of the study community through two years postpartum (all participants). Exclusion criteria included inability to undergo study measurements due to mental (eg, active psychosis or severe claustrophobia) or physical condition (e.g., weight >250 kg that would prevent the use of study equipment) as well as any current treatment for any form of diabetes mellitus or hypertensive disorder based on participant self-report and medical record review.

### Recruitment and enrollment

The study enrolled approximately 1920 pregnant women ≤18 weeks GA (804 WHIV, 1116 HIV-seronegative) from September 2021 to December 2023. Potentially eligible women were identified at their 1^st^ antenatal care visit and those who were interested in study participation were referred to a University of Cape Town research facility located at the Gugulethu Community Health Centre where enrollment and all study visits and procedures are conducted. Prior to the initiation of any study activities, all pregnant women underwent a written informed consent process. After delivery and prior to any postpartum visit procedures, enrolled participants are also undergoing a re-consenting process at which time women are asked to provide written informed consent for participation of newborn infant. As of March 2024, participant follow-up and data collection activities are ongoing (for both women and their infants). The last delivery is expected to occur in July 2024 at which time the infant cohort will be fully enrolled. The last expected participant visit will take place in July 2026.

### Ethics approval

The ORCHID study protocol was reviewed and approved by the Faculty of Health Sciences Human Research Ethics Committee of the University of Cape Town (UCT-HREC 709/2020), and Institutional Review Boards (IRBs) of Columbia University Irving Medical Center (AAAT6112) and the Ann & Robert H. Lurie Children’s Hospital of Chicago (2021–4725) in the USA.

### Inclusivity in global research

Additional information regarding the ethical, cultural, and scientific considerations specific to inclusivity in global research is included in the Supporting Information (S3 File).

### Primary exposures of interest

Enrollment was distributed across three main exposure categories by HIV status and DTG use (hereafter, HIV/DTG exposure category): (i) WHIV initiating DTG-based ART during pregnancy (iDTG); (ii) WHIV already on DTG-based ART before enrollment and continuing DTG through pregnancy (cDTG); and (i) women without HIV infection (HIV-). HIV infection status is confirmed at enrolment, and HIV antibody testing in women without HIV is conducted at each study visit to detect incident HIV infections [34].

### Primary outcomes and key study assessments

Study assessments for maternal and child participants as well as the timing of each are summarized in Fig 1 where primary outcomes are indicated in bold. Table 1 summarizes key measures and sample size for the MC and each nested sub-study. At each study visit, participants complete a series of questionnaires, anthropometry and blood pressure are measured and laboratory specimens (blood and urine) are collected. Additional measures include obstetric and hepatic ultrasounds, oral glucose tolerance testing (OGTT), indirect calorimetry, and air displacement plethysmography (ADP). Umbilical cord blood is collected at delivery and breastmilk samples are collected during the first months of life. Fat biopsies are obtained from a subset of women enrolled in the nested sub-study. Child participant follow-up includes additional questionnaires, anthropometry, laboratory sampling (blood only), and ADP. Clinical and laboratory data are abstracted from routine medical records for all participants.

#### Maternal assessments and outcomes: Main cohort

*Locally-established questionnaires* are employed to measure demographics and health status, family and household composition, economic activity, HIV disease including history of HIV-related conditions and ART use including previous ART use, duration of current ART, and ART adherence [35, 36]. Physical activity is assessed using the International Physical Activity Questionnaire (IPAQ) [37] and the Pregnancy Physical Activity Questionnaire (PPAQ)) [38]. Food and caloric intake are measured via 7-day and 24-hour recall using the Western Cape Food Frequency Questionnaire (WC-FFQ), a locally developed instrument whose validation included data from the Gugulethu community [39]. Using the South African (SA) Medical Research Council’s Foods Composition Database [40], WC-FFQ responses are converted to estimate energy, carbohydrate, protein, fat, and fiber intake based on 1741 commonly consumed foods in South Africa. Other measurements include: satiety and appetite [Three-Factor Eating Questionnaire (TFEQ)] [41, 42]; HIV stigma (Internalized AIDS-Related Stigma Scale) [43] and obesity stigma [44–47]; food access [48, 49] and food insecurity [50]; smoking [Alcohol, Smoking and Substance Involvement Screening Test (ASSIST)] [51], alcohol use [Alcohol Use Disorders Identification Test (AUDIT)] [52] and drug use [Drug Use Disorders Identification Test (DUDIT)] [53]. In additional the study is investigating a broader range of psychosocial constructs that may play a role in shaping maternal and child health and associated behaviours, including: unintended pregnancy [London Measure of Unplanned Pregnancy (LMUP)] [54]; experiences of interpersonal violence (WHO Violence Against Women questionnaire) [55]; sleep quality [Pittsburgh Sleep Quality Index (PSQI), Berlin Questionnaire)] [56, 57]; adverse childhood experiences [WHO Adverse Childhood Experiences International Questionnaire (ACES)] [58]; experiences of community violence [59]; and access to social support [60]. Depression is measured using the Edinburgh Postnatal Depression Scale (EPDS) [61] and the Patient Health Questionnaire (PHQ-9) [62] while anxiety is measured using the General Anxiety Disorder Questionnaire (GAD-7) [63].

Additional clinical information is obtained via Medical Record abstraction including HIV testing history, maternal HIV history and ART use, past and current obstetric history, intercurrent medical history including medications, hospitalizations, concomitant medications, and family planning including contraception. ART adherence is obtained via self-report and from pharmacy records using approaches the team has validated previously in this setting [64–67]. HIV viral load (VL) is measured every three to six months among WHIV.

*Anthropometry* includes height (via stadiometer) and weight (for BMI calculation), middle-upper-arm circumference, and skinfold thicknesses with calipers (Harpenden skinfold calipers) (triceps, subscapular, suprailiac) for adiposity estimation. For each, three measurements are performed, and readings were averaged.

*Blood pressure* is measured using automated monitors (Edan M3A, Medline Industries). Participants are seated upright and rested for a minimum of 5 min. With the right arm supported, three measurements are performed 1 min apart using appropriately sized cuffs.

*Adipose tissue mass* is measured by ADP [68], using the non-invasive BodPod system (Cosmed, USA). This system uses whole-body densitometry to determine fat and fat-free mass in an assessment lasting 3–5 minutes. ADP has been widely validated in adults, including the context of HIV infection and pregnancy, with excellent acceptability and accuracy [30, 69–75]. Per our prior research established hydration constants specific to late pregnancy are used, and thoracic volume is measured [30, 73, 75].

*Resting energy expenditure* (REE) is measured via indirect calorimetry, at ≤18 and 32–36 weeks GA using the Q-NRG system (CosMed, Rome, Italy), a widely used research measure for REE validated across a range of BMI and disease states [76, 77]. The Q-NRG measures respiratory volumes, oxygen consumption, and CO_2_ production at 30-second intervals to calculate REE using the abbreviated Weir equation and a fixed respiratory quotient.

*Dysglycemia and Insulin Resistance (IR)* are assessed using a 75g oral OGTT with insulin, glucose and C-peptide measurements collected at 0-, 30-, 60-, and 120-minutes post OGTT after a 10-hour overnight fast. Glucose, insulin, and c-peptide tests are conducted at the SA- National Health Laboratory Services (NHLS) in Groote Schuur Hospital in Cape Town. First-phase insulin secretion, clearance and β-cell function are measured using the *Mari model* [78–80]. Thereafter, insulin sensitivity is assessed using the *Matsuda Index* [81] which has been validated in pregnancy [82]. These measurements are then used to calculate the *Oral Disposition Index (DIo)* which provides an accurate measure of insulin secretion relative to the amount of insulin sensitivity as well as pancreatic beta cell function; DIo values of <1.24 have been shown to predict future diabetes mellitus (DM) risk [83]. Dysglycemia is defined as impaired fasting glucose or impaired glucose tolerance [84]. Gestational diabetes mellitus (GDM) is defined as meeting any one of the following glucose (mg/dL) criteria: fasting ≥92, 1h ≥180, or 2h ≥153 [85].

*Dyslipidemia* is assessed using a fasting lipid profile (high-density lipoprotein [HDL], low-density lipoprotein [LDL], triglycerides [TG]).

*Hepatic steatosis* is assessed with ultrasound using scored B-mode imaging operated by a trained research ultrasonographer [86–88].

#### Fetal/Child assessments and outcomes: Main cohort

*Gestational age and fetal growth* are measured via obstetric ultrasound. Standard fetal biometry is used for pregnancy dating and for monitoring intrauterine growth [89].

*Locally-established questionnaires* are employed for the assessment of congenital anomalies, intercurrent medical history including concomitant medication use, breastfeeding status/history, infant and child caloric intake in the past 24 hours [39, 40], childcare [90], feeding practices [Comprehensive Feeding Practices (CFPQ)] [91] and neurodevelopment (WHO Global Scale for Early Development tool) [92]. Medical records are abstracted to obtain delivery details and birth outcomes [birthweight, gestational age, congenital anomalies [93]], vaccination history, HIV testing history, intercurrent medical history including ambulatory presentations (such as respiratory or diarrheal illnesses), medications, and hospitalizations.

*Anthropometry* includes the assessment of weight, length (via stadiometer), head and waist circumferences, and skinfold thicknesses with calipers (Harpenden skinfold calipers) (triceps, subscapular, suprailiac) for adiposity estimation. For each, three measurements are performed and readings were averaged.

*Adipose tissue mass* is measured by ADP [68], using the PeaPod system (Cosmed USA). This system uses whole-body densitometry to determine fat and fat-free mass in an assessment lasting 3–4 minutes. The team has previously validated ADP from one week to 6 months of age or 8kg, and for both premature and term infants [94–96].

*Dysglycemia and IR* are examined using insulin and glucose (for Homeostasis model assessment of insulin resistance [HOMA-IR] estimation) collected after a standardized meal and fast, and *dyslipidemia* is examined using pre-prandial lipids [TG, HDL, LDL].

#### Maternal assessments and outcomes: Nested sub-studies

*Systemic and adipose inflammation*, *gut integrity and satiety/hunger markers*: Using stored plasma from ≤18 and 32–36 weeks GA, retrospective testing will be conducted a subset of participants to assess markers of systemic and adipose inflammation (sCD163, sCD14, interleukin-6 [IL-6], leptin, adiponectin), gut integrity (intestinal fatty acid binding protein [I-FABP]), and satiety/hunger (alpha melanocyte stimulating hormone [α-MSH], ghrelin) using enzyme-linked immunosorbent assays (ELISAs) (Abcam, Cambridge, MA).

*Subcutaneous Adipose Tissue (SAT) function* will be measured following a 10-hour overnight fast via 1-2g SAT biopsy (buttock) performed by a trained surgical medical officer with local lidocaine administration. SAT specimens are assessed for morphology and histology by an histopathologist blinded to participant information. The mitochondrial function of SAT, as measured by high-resolution respirometry, is assessed in real-time using Oxygraph (Oroboros Instruments Corp, Innsbruck, Austria), and the remaining samples are snap-frozen in liquid nitrogen and stored at -80°C in ~100 mg aliquots for future analyses of gene expression, protein levels, and metabolomic/lipidomic analyses.

*Metabolites and lipid subspecies*. Metabolomic profiling uses ~1.0 mL of plasma from each participant collected at ≤18 and 32–36 weeks GA and stored at -80°C. Liquid chromatography mass spectrometry (LCMS/MS) developed for the Sciex 6500+ QTRAP system will provide comprehensive data on approximately 600+ small metabolites, 1300 lipid species in 26 lipid classes, and ~100 distinct eicosanoids.

#### Fetal/Child assessments and outcomes: Nested sub-studies

*Metabolites and lipid subspecies*. Infant metabolomic profiling uses ~1.0 mL of plasma from cord blood collected at birth and stored at -80°C before shipping to the Albert Einstein College of Medicine Metabolomics Core where metabolomics and lipidomics techniques similar to that described above will be utilized.

*Breastmilk composition and DTG exposure*. Metabolomic profiling of breastmilk specimens parallel the measures described above, comprehensively identifying fatty acids and their distribution among lipid species, as well as additional analytes in milk that may be susceptible to the maternal metabolism. DTG levels will be measured in both breastmilk and infant plasma at the infant 6 weeks visit using LCMS [97, 98].

### Data management and analysis

#### Data collection and management

Data are collected on tablets linked to a RedCap database developed for this study, with paper forms used for meta-documentation (e.g. specimen logs) as well as a backup in the event of technical failure. Externally derived data (such as from laboratory assays) is imported using pre-specified templates. Data merging and cleaning, including quality control (QC) reporting, are completed weekly. After completion of the study, de-identified datasets will be prepared and made available to the NICHD Data and Specimen Hub (DASH), a centralized resource for researchers to store de-identified data and to access data and associated biospecimens from NICHD-supported studies for use in secondary research.

#### Sample size

The sample size for the MC is based on Aim 2, to test the association of HIV/DTG with postpartum metabolic health. The clinically-driven cutpoint of DIo<1.24 was used as this is strongly associated with an increased risk of subsequent diabetes within 10 years [83]. We assumed a 2-sided test at β = 0.1 (90% power), a 1:2 ratio of HIV+ to HIV- women, and a background prevalence of DIo<1.24 of 2% at 2 years PP based on our prior research in this setting [99]. Further, we assumed 87% cohort retention from enrollment at ≤18 weeks GA through 2 years PP, 6% participant withdrawal for medical reasons (including pregnancy losses, maternal/infant death, and <2% incident HIV in mothers or infants through 24m PP) and 2% censoring due to ART switches among WLHIV on DTG, all from previous studies [34, 100–104]. Based on this, we estimated that n = 1200 women will be required to detect absolute increases in DIo<1.24 of 4% (to levels of 6% among WLHIV at 2 years PP) for Aim 2; from this, we estimated enrolling up to 1900 pregnant women into the study. When DIo is considered as a continuous variable, this will provide >90% power to detect mean DIo differences of 0.5 units, against a background value of 2.6 in women 25–34 years old in SA (SD = 1.6) [99]. In addition, this cohort size provides an adequate sampling frame for all sub-studies.

#### Analysis

Data analyses will use R (R Foundation, Vienna, Austria). Throughout, descriptive and bivariable statistics will follow standard approaches. Person-time in prospective analyses will be included as long as women remain in their initial exposure categories, with censoring due to incident HIV infection or changes to ART regimens, in addition to death or loss to follow-up. Appropriate distributional assumptions will be examined before deploying specific methods, with alternate approaches considered when required. The potential for missing data to influence findings will be assessed through sensitivity analyses using multiple imputation with chained equations [105]. For primary outcomes, hypothesis testing will use 2-sided tests with family-wise error rate control [106–108]; exploratory analyses will use false discovery rate corrections (Benjamini-Hochberg method; α = 0.05) [109, 110]. Generalized linear mixed models (GLMM) will be used extensively to examine associations between HIV/DTG exposure category and various outcomes of interest, using random effects to account for repeated measures (in our cohort designs including the MC) and/or repeated observations linked to our designs (in case-cohort designs).

## Discussion

Using a multi-disciplinary approach with efficient case-cohort “ambi-directional” designs to examine the intersection of maternal, fetal, and child health, ORCHID leapfrogs traditional long timelines in investigating the safety signals in mothers and their children by studying DTG in pregnant WHIV in South Africa where obesity and DTG use are rapidly escalating. Integrating mechanistic, clinical, and epidemiologic studies, this research will develop a novel conceptual framework for evaluating the effects of other obesogenic agents, an important advance given the increasing use of obesogens in pregnancy and breastfeeding.

With ORCHID fully accrued, potential challenges may include commonplace retention concerns, particularly given the large sample size and the burden of study visits and time- consuming evaluations. However, our extensive experience in retaining mother-infant pairs in research in this setting has led to detailed methods for minimizing cohort attrition over time. Detection biases are possible for self-reported measures, but the major study measures are objective and/or blinded and unlikely to be subject to systematic errors. Distinguishing the effects of HIV disease from DTG use is complex, but we have included separate groups of women initiating and continuing DTG and included regular measures of VL over time to help address this, in addition to including an HIV- comparison group. Since, most adults including pregnant women globally are currently on a DTG-based regimen it would be impossible to include a comparison group of pregnant women receiving a non-DTG-based ART. Finally unexpected events including political unrest, climate hazards, and infectious outbreaks have the potential to disrupt enrollment and follow-up; however, having initiated the study during the COVID-19 pandemic, the study team and participants have experience adapting to adverse conditions.

The results of this study will advance our understanding of the obesogenic mechanisms and clinical and metabolic consequences of DTG in pregnant and postpartum women with HIV and their offspring, moving the field of maternal-fetal metabolic health forward in the HIV arena, and as a result, inform the next generation of intervention studies to reduce the chronic comorbidities of HIV and ART over the life course for WHIV and their children.

## Supporting information

S1 File ORCHID protocol. (PDF)

S2 File SPIRIT checklist. (PDF)

S3 File Inclusivity in global research checklist. (PDF)
