# Supplementary material for: An observational cohort study to investigate the impact of dolutegravir in pregnancy and its obesogenic effects on the metabolic health of women living with HIV and their children: Study protocol
Source: PLoS One. 2024 Aug 19;19(8):e0307296. doi: 10.1371/journal.pone.0307296 (PMC11332920; doi:10.1371/journal.pone.0307296)
Supplement: S1 File — (PDF) [file pone.0307296.s001.pdf]

Protocol Title:

Obesogenic origins of maternal and child metabolic health involving dolutegravir (ORCHID)

Principal Investigators:

Elaine Abrams (Pediatrics & Epidemiology, Mailman School of Public Health, ICAP Columbia University)
Jennifer Jao (Paediatrics & Medicine, Feinberg School of Medicine, Northwestern University /Lurie Children's Hospital)
Landon Myer (Epidemiology & Biostatistics, University of Cape Town)

Co-Investigators:

Irwin Kurland (Endocrinology, Department of Medicine, Albert Einstein College of Medicine)
Patrick Catalano (Obstetrics and Gynecology, School of Medicine, Tufts University)
Grace McComsey (Pediatric Infectious Diseases, School of Medicine, Case Western Reserve University)
Mariana Gerschenson (Department of Medicine, Mānoa (UHM) John A. Burns School of Medicine, University of Hawaii)
Julia Goedecke (Human Biology, University of Cape Town & SAMRC)
Jami Josefson (Paediatrician, Feinberg School of Medicine, Northwestern University /Lurie Children's Hospital)
Daniel Robinson (Neonatology, Feinberg School of Medicine, Northwestern University /Lurie Children's Hospital)

Investigator's expertise/specialties:

Investigator	***Expertise***
**Landon Myer *(mPI)***	HIV in maternal/women's health; epidemiologic methods
**Elaine Abrams *(mPI)***	HIV/ART in mothers & children; scientific leadership
**Jennifer Jao *(mPI)***	Metabolic complications of HIV in mothers & children
**Irwin Kurland**	Metabolomic methods & HIV-related pathways
**Patrick Catalano**	Obesity in pregnancy: measurement, sequelae
**Grace McComsey**	HIV, inflammation and obesity in women and children
**Mariana Gerschenson**	Adipocyte and mitochondrial function in HIV/ART
**Julia Goedecke**	Obesity in SA women: mechanisms, measurement
**Jami Josefson**	Neonatal/infant obesity & metabolic complications
**Daniel Robinson**	Breastmilk composition and child health

Funding: National Institutes of Health (1R01HD104599-01)

A. PURPOSE AND STUDY AIMS

More than 8 million people are living with HIV in South Africa (SA), including >250,000 women who become pregnant annually, and >50% of SA women are overweight/obese.[1] In SA and globally, Dolutegravir (DTG)-based antiretroviral therapy (ART) is being scaled up as part of the preferred 1st-line ART regimen. However, DTG has recently been implicated as an obesogen that is associated with increased weight and adipose tissue gain compared to other antiretroviral agents.[2,3] Obesity in pregnancy is associated with poor health outcomes for both mother and child[4-9] as pregnancy is a critical period during which exposures leading to alterations in metabolic health may influence not only long-term maternal health but also fetal, neonatal, and ultimately child health. For women living with HIV (WLHIV) and their children, these exposures are myriad, including HIV/ART, weight gain, & obesity. Despite this, there is a paucity of data on DTG and its obesogenic effects in pregnant WLHIV and their children.

To address this gap, the **central objective of this proposal is to investigate the impact of DTG in pregnancy and its obesogenic effects on the metabolic health of women living with HIV (WLHIV) and their children, compared to women without HIV and their children.** Our team of leading researchers from the fields of HIV in pregnancy; metabolic complications in HIV/ART; obesity and its sequelae in pregnant women and their children; and health of HIV-exposed uninfected infants is well positioned to address this objective.

Overview: We will enroll up to 1900 pregnant women in the 1st trimester and their children, following them to two years. As part of this, mother-infant pairs will be required to attend up to 10 study visits separate from routine clinic visits, these visits include 3 antenatal visits (≤18, 24-28 and 32-36 weeks) and 7 postnatal visits (<2 and 6 weeks, 3, 6, 12, 18 and 24 months). Measurements in **mothers** will include demographics and health status, HIV disease and ART use, intercurrent medical history including concomitant medication use, HIV viral load testing, ART adherence, HIV antibody testing in women without HIV; body composition, caloric intake, dysglycemia and insulin resistance (IR), lipid profiles, anthropometry, resting energy expenditure, hepatic steatosis, specimen collection (whole blood, plasma, serum, placenta and breastmilk), systemic and adipose inflammation, as well as metabolites, lipid subspecies and eicosanoids. Measurements in **infants** will include uterine gestational age and fetal growth, as well as metabolites, lipid subspecies and eicosanoids, body composition, dysglycemia and IR, lipid profiles, anthropometry, feeding, specimen collection (cord blood, whole blood, plasma and serum) and intercurrent medical history including concomitant medication use. Additional data on maternal health in pregnancy and birth outcomes will be abstracted from medical records.

The **specific aims are to:**

Aim 1: Examine how HIV and/or DTG use (HIV/DTG) impact longitudinal changes in weight and adipose tissue mass in pregnancy in a population where HIV and obesity are highly prevalent.

- **Aim 1a:** Investigate whether imbalances between caloric intake and resting energy expenditure (REE) are associated with longitudinal changes in weight and adipose tissue mass in pregnancy among WLHIV receiving DTG.
- **Aim 1b:** Investigate whether markers of systemic and adipose inflammation, gut integrity, and satiety/hunger are associated with longitudinal changes in weight and adipose tissue mass in pregnancy among WLHIV receiving DTG.

Aim 2: Examine the association of HIV/DTG use with maternal metabolic health postpartum.

- **Aim 2a:** Investigate the association of longitudinal changes in weight and adipose tissue mass in pregnancy with metabolic health postpartum in WLHIV receiving DTG.
- **Aim 2b:** Investigate whether a signature cluster of metabolites and lipid subspecies in pregnancy are associated with metabolic health postpartum in WLHIV receiving DTG.

Aim 3: Examine the association of *in utero* exposure to HIV/DTG with neonatal and child metabolic health through two years of life.

Aim 3a: Investigate the association of neonatal weight and adiposity with neonatal and child metabolic health in HIV/DTG-exposed children.
Aim 3b: Investigate whether a signature cluster of metabolites and lipid subspecies in cord blood are associated with neonatal and child metabolic health in HIV/DTG-exposed children.
Aim 3c: Explore the associations of breastmilk composition, DTG exposure, and child metabolic health in HIV/DTG-exposed children.

Impact: This comprehensive set of studies investigate maternal DTG use and adiposity in pregnancy, and its downstream effects on both maternal postpartum and neonatal/child metabolic health. The results of our study will provide insights into metabolic disease risk reduction in the context of HIV/ART, identify potential targets for interventions, and inform public health approaches to diminish chronic co-morbidities over the life course for WLHIV and their children.

B. BACKGROUND, SIGNIFICANCE AND PRELIMINARY DATA

Worldwide obesity: impact on life course health/morbidity. Almost 2 billion adults and 41 million children are overweight/ obese.[10] The global obesity epidemic now includes middle-income countries such as SA where 54% of adults are overweight/obese.[11] Obesity increases diabetes and cardiovascular disease (CVD) risk,[12] lowering quality of life[13] and life expectancy.[14]

HIV and antiretrovirals (ARV): from infectious to chronic sequelae. In SA there are >6 million adults on antiretroviral treatment (ART), >55% of whom are women of reproductive age; almost 20% of all pregnant women are living with HIV.[15] The success of widespread ART is indisputable, but chronic co-morbidities such as obesity, diabetes, and CVD, associated with HIV/ART, are increasingly being recognized. In SA, the United States (US), and other settings, we are witnessing the collision of the obesity and HIV epidemics.

Pregnancy: a critical window shaping chronic disease risk. A life course approach to chronic diseases posits that various exposures at pivotal stages in life trigger differential lasting consequences on the long-term health of an individual.[16] Pregnancy is a critical period during which exposures leading to alterations in metabolic health may influence not only long-term maternal health but also fetal, neonatal, and ultimately child health. For WLHIV and their children, these exposures are myriad, including HIV/ART, weight gain, & obesity.

Dolutegravir (DTG): an obesogenic ARV. DTG, a potent, well-tolerated integrase strand transfer inhibitor (INSTI), has been used widely in the US since 2015. In 2019 the World Health Organization recommended DTG with tenofovir and lamivudine (TLD), given as one pill once daily, as the preferred 1st-line regimen for all persons living with HIV (PLHIV) including pregnant women.[17-19] It is a global priority to scale-up TLD in countries heavily affected by HIV, including SA, with projections that 15-20 million individuals and 5-8 million pregnant WLHIV will receive TLD over the next 3-5 years.[20] Recent studies demonstrated that compared to other ARV classes, INSTIs are associated with greater weight and waist circumference increases[21-26] as well as hyperglycemia[27] in both PLHIV initiating ART and those switching regimens. In the NA-ACCORD study, participants initiating DTG or raltegravir (RAL), another INSTI, gained significantly more weight at 2 and 5 years post initiation vs. those initiating non-nucleoside reverse transcriptase inhibitor (NNRTI)-based ART.[22,28] The ADVANCE trial in SA revealed higher weight and fat gain in those initiating DTG- vs efavirenz (EFV)-based ART; this finding was strongest in women.[2,3] Our studies and others have demonstrated DTG's transplacental transfer and detection in breastmilk.[29,30] Despite this, there is a paucity of data on DTG and its obesogenic effects in pregnant WLHIV and their children.

Gestational weight gain (GWG): a window into the metabolic health of women and their offspring. Excess GWG is associated with preeclampsia, gestational diabetes (GDM), and long-term adiposity and obesity.[6-9,31-34] Furthermore, we have shown that women with excessive vs. adequate or inadequate GWG had greater adipose mass increase (5.2 vs. 0.2 and -2.7 kg respectively, $p<0.001$).[35] The risk of postpartum (PP) weight retention has been reported to be 2-fold higher in women with excessive GWG.[36] In addition to PP weight retention[36] and increased abdominal adiposity later in life,[34] women with excessive GWG have a 47% higher risk of developing diabetes over 20 years PP compared to those with appropriate GWG.[37] Furthermore, we and others have demonstrated that women with excessive early GWG are more likely to have infants with higher birth weight, adiposity,[38-40] and fetal IR.[38,39,41]

DTG impacts pregnancy and PP weight. Both the Tsepamo study in Botswana and the IMPAACT VESTED study comparing the efficacy and safety of DTG vs. EFV-containing ART reported higher GWG in WLHIV receiving DTG vs. EFV.[42,43] Our preliminary data (MCH-ART: 451/2012; PIMS: 739/2014; BPOS: 541/2015) have also demonstrated <u>very high levels of overweight, obesity and GWG among pregnant WLHIV in SA</u>. In MCH-ART, 43% of pregnant women had BMI>30 kg/m²;[44] and in PIMS, 25% of women had GWG exceeding Institute of Medicine (IOM) norms.[45] In addition, we observed that <u>PP weight gain is associated with DTG use</u>. Our Tshilo Dikotla study (IRB 2019-2922) demonstrated that WLHIV receiving TLD had higher weight through 18 months PP vs. those on tenofovir/ lamivudine/efavirenz (TLE),[46] and data from our randomized trial of TLD vs. TLE initiated in pregnancy (DolPHIN2) showed DTG was associated with continued weight gain to 48 weeks PP[47] (Fig 1).

Figure 1. Postpartum weight over time comparing DTG vs EFV in DoLHPIN2 & Tshilo Dikotla

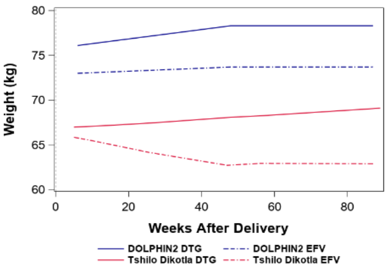

Limited data on caloric intake and resting energy expenditure (REE) in WLHIV and pregnancy. Weight-adjusted REE and diet both impact total energy expenditure and GWG in African-American women in pregnancy.[48] We have shown that women with smaller increases in REE also had greater GWG, and REE decreases with fat mass (rho= -0.27, p=0.06).[49] However, there are few data on caloric intake and REE in pregnant African women.[50] <u>Data on caloric intake and REE in PLHIV is also sparse and poorly understood</u>.[51] We showed higher total energy expenditure in WLHIV on protease inhibitors (PI) or NNRTIs vs. women without HIV,[52] but others have not.[53,54] Further, data differ on whether HIV viral suppression promotes lower REE,[52,55,56] highlighting the need to understand caloric intake and REE in the context of DTG use and pregnancy.

Adipose inflammation, gut integrity, and satiety/hunger influence weight gain and adipose accrual in PLHIV. Few data exist on adipokines in pregnancy,[57,58] though we have previously shown pregnancy to be associated with a leptin-resistant state.[59] Obesity is associated with chronic low-grade inflammation in adipocytes manifested by increased activated macrophages in adipose tissue expressing cytokines including interleukin-6 (IL-6), tumor necrosis factor-alpha (TNF-α)[60,61] and higher levels of soluble (s)CD163.[62,63] Among PLHIV, obesity is independently associated with elevated IL-6, sCD163, and C-reactive protein.[64] Despite an overall improved metabolic profile with INSTIs, we showed that WLHIV who switched to RAL from PI or NNRTI-based ART had persistently elevated sCD163 and intestinal fatty acid binding protein (I-FABP), a marker of gut integrity.[65] Furthermore, we have shown that a 2-fold increase in I-FABP before ART start is associated with a 9% increase in total adipose gain over 96 weeks of ART,[66] suggesting the role of inflammation and gut integrity with adipose accrual in PLHIV on ART. Few studies have examined markers of adipose inflammation, gut integrity, and satiety/hunger in the context of DTG; recent studies report conflicting findings regarding DTG's effect[67,68] on alpha melanocyte

stimulating hormone (α-MSH) binding to melano-cortin-4 receptor (MC4R), a pathway driving increased food intake, leptin levels, and severe obesity.[69,70]

Understanding the maternal PP and fetal response to a unique maternal pregnancy milieu. Several studies demonstrate that maternal metabolic health in pregnancy has implications for long-term maternal and child metabolic health.[85-89] We showed that maternal BMI, weight gain, and glucose metabolism in pregnancy all influence women's risk for glucose metabolism disorders after pregnancy as well as neonatal/child adiposity and insulin sensitivity in populations without HIV in the HAPO[90-93] and PANDORA studies.[4] However we do not know the impact of maternal HIV and exposure to an obesogen (DTG) in pregnancy on fetal metabolic programming, and maternal PP and child metabolic health.

A unique maternal milieu: HIV, an obesogen (DTG), obesity, higher 1st phase insulin secretion, and higher SAT. In normal pregnancy, as gestation progresses, so too does IR, increasing by 50% by 3rd trimester (T3).[94] Our preliminary data from healthy pregnant women reveals that 1st phase insulin secretion is lower in T3 than 1st trimester (T1) (Fig 2). Interestingly, we found pregnant WLHIV on TLD to have lower risk for GDM than those receiving TLE (adjusted OR: 0.40, 95% CI: 0.18-0.92; 6.1% with TLD vs. 13.5% with TLE). This paradox of lower GDM risk but increased weight gain with DTG could be explained by our hypothesis: WLHIV on DTG have higher 1st phase insulin secretion in T3 compared to healthy pregnant women (Fig 2).[95] Higher 1st phase insulin secretion is associated with increased hepatic lipogenesis, increased adipose uptake of fatty acids (FAs), and decreased lipolysis, resulting in peripheral adipose accumulation.[96] The increased PP weight gain we observe in WLHIV on DTG may be explained by this adipose accumulation associated with higher 1st phase insulin secretion (Fig 3).[97,98] Higher 1st phase insulin secretion at the expense of adipose gain (higher SAT) may be a useful metabolic adaptation in pregnancy with DTG, particularly if this prevents hepatic steatosis. However, there are no data on hepatic steatosis in WLHIV using DTG or the effect of these adaptions on long-term maternal and child metabolic outcomes.

Figure 2. Insulin secretion in normal pregnancy by trimester and hypothesized effect with DTG

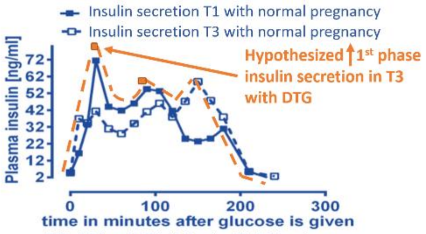

DTG=dolutegravir; T1=1st trimester; T3=3rd trimester

Figure 3. Proposed pathways involved in the establishment of a unique maternal milieu by HIV/DTG in pregnancy, the fetal response to this unique maternal milieu, and downstream impacts on both maternal postpartum and child metabolic health

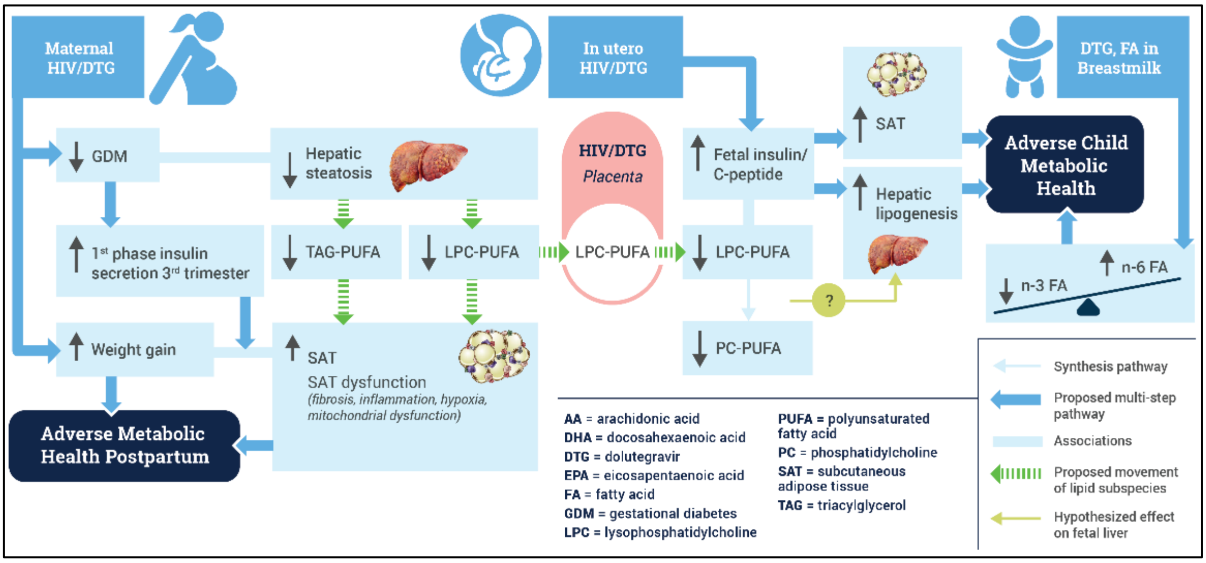

An altered maternal metabolome in the face of HIV, DTG, and African ancestry. Our preliminary data comparing PLHIV enrolled in the ACTG 5260s protocol and age, sex, race, and BMI-matched adults without HIV from the MACS/WIHS cohorts show that there are distinct metabolomic signatures between individuals without HIV and PLHIV on RAL (Fig 4). Metabolites driving these differences were lower levels of long-chain triacylglycerol (TAG)-polyunsaturated fatty acids (PUFAs) (TAG-PUFAs) and lysophosphatidylcholine (LPC)-PUFAs (LPC-PUFAs) in PLHIV on INSTI-based ART, suggesting lower hepatic steatosis. This is opposite to what we observed in PLHIV on PI-based ART which is associated with higher TAGs of shorter chain length and lower levels of unsaturation which may signify hepatic lipogenesis and steatosis in those on PIs. The metabolite signature in PLHIV on INSTI-based ART supports our hypothesis that WLHIV on DTG maintain insulin sensitivity and first phase insulin response and take up excess TAG-PUFAs into accumulating SAT, which acts as a 'sink', protecting against hepatic steatosis (Fig 3). We showed that outside of pregnancy, diabetes in black SA women is associated with hyperinsulinemia and higher SAT but lower hepatic and visceral fat, an adipose distribution that is vastly different from white women in North America/Europe.[82] This distribution would appear beneficial in some populations but is, in fact, associated with IR in Africans.[79,82] Further, while potentially advantageous in pregnancy, SAT accumulation PP may result in an inflammatory environment with eventual oxylipid/eicosanoid production and fibrosis, eventually leading to ectopic fat deposition including hepatic steatosis. This highlights the importance of elucidating DTG's role in metabolic dysregulation during and beyond pregnancy.

The fetal response to a unique maternal metabolic milieu in the context of HIV and DTG. Lower levels of maternal circulating TAG-PUFAs and LPC-PUFAs, increased SAT accumulation, and higher 1st phase insulin secretion through T3 gestation, all associated with HIV and DTG, creates an altered *in utero* environment to which the fetus must respond. LPC-PUFAs (carriers for PUFAs across the placenta), are transferred to the fetal circulation via transporter proteins, including the major facilitator superfamily domain containing 2A (Mfsd2A).[99] Lower maternal LPC-PUFAs likely translate into lower LPC-PUFAs in HIV/ARV-exposed uninfected (HEU) fetuses and newborns (Fig 3). This may promote higher fetal lipogenesis since higher circulating PUFAs are associated with suppression of lipogenesis.[100,101] We have already observed lower overall cord PUFAs as well as PC-PUFAs, (downstream products of LPC-PUFAs) as a distinct pattern distinguishing HEU vs. HIV-unexposed uninfected (HUU) neonates.[102] We also found higher levels of cord insulin and C-peptide, in HEU vs. HUU neonates, after adjustment, reflecting higher IR in HEU neonates (Table 1). This combination of lower cord PUFAs and higher insulin is similar to findings in cord blood of neonates of mothers with GDM[103] and is provocative, warranting further investigation, since our data in fact, show WLHIV on TLD to have lower risk for GDM. In addition, we noted PUFAs and IL-6 were associated with cord C-peptide in HEU but not HUU neonates, raising the possibility that fetal metabolic programming occurs in response to *in utero* HIV/ARV exposure; higher fetal insulin and C-peptide suggests increased fetal adipose accumulation.[91,92] We also found distinct signatures of cord eicosanoid metabolites, with higher pro-inflammatory [derivatives of arachidonic acid (AA), an n-6 PUFA] and lower anti-inflammatory [derivatives of eicosapentaenoic acid (EPA), an n-3 PUFA] eicosanoids in HEU vs. HUU neonates,[102,104] suggesting an inflammatory intrauterine environment. Taken together, we hypothesize the fetal response to the unique maternal metabolic milieu of pregnant WLHIV on DTG results in IR, adiposity, and inflammation in childhood.[90]

Figure 4. Group separation of metabolites in PLHIV on RAL-based ART vs. HIV- individuals by orthogonal partial least squares discriminant analysis

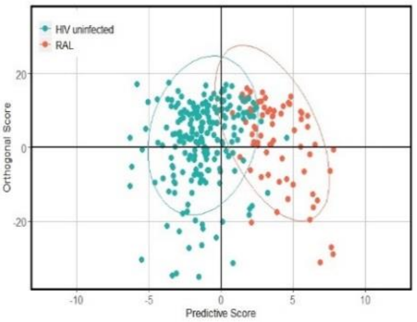

Table 1. Associations of *in utero* HIV/ARV exposure with Cord Blood Insulin and C-peptide

Model Outcome	*Coefficient*	*p*
Log Insulin	0.295	0.03
Log C-peptide	0.522	<0.01

Neonatal and child metabolic health differs by *in utero* HIV/ARV exposure. We demonstrated altered fuel utilization and intermediary metabolites in HEU infants in Cameroon.[105] We found in Botswana that compared to HUU infants, DTG-exposed HEU infants exhibit higher Homeostatic Model Assessment IR (HOMA-IR) from birth to 2 years (Table 2). Additional preliminary data in our US cohort shows higher low-density lipoprotein (LDL) and total cholesterol (TC) in HEU vs. HUU children at 1 year (Table 3), underscoring the need to assess metabolic health into childhood.

Table 2. HOMA-IR at Birth, 1m & 24m in HEU and HUU infants

	HEU infants	HUU infants	*P*
Birth (n=379)	0.38 (0.23-0.61)	0.29 (0.20-0.55)	0.012
1m (n=399)	0.76 (0.46-1.21)	0.62 (0.39-0.99)	0.010
24m (n=99)	1.28 (1.09-2.00)	1.13 (1.03-1.67)	0.044
	In *utero* DTG/HIV-exposed infants	HUU infants	*P*
Birth (n=272)	0.35 (0.24-0.57)	0.29 (0.20-0.55)	0.055
1m (n=279)	0.73 (0.44-1.13)	0.62 (0.39-0.99)	0.053
24m (n=76)	1.26 (1.11-1.54)	1.13 (1.03-1.67)	0.070

Breastmilk FAs contribute to child metabolic health. Maternal overweight/obesity alters the content of breastmilk FAs, regulators of infant adiposity. In conditions of maternal overweight/obesity as opposed to normal weight, colostral and mature milk's n-6:n-3 (omega 6: omega 3) FA ratio increases 10-16%.[106,107] An 1-unit increase in the ratio of n-6:n-3 FA in milk predicts increasing percent body fat (β=4.7%) in US infants at age 4 months,[108] and increased fat mass gain during the first 4 months.[109,110] Linoleic acid (LA) is the most highly concentrated n-6 FA in breastmilk. Our pilot data identified a strong correlation between linoleic acid and pre-pregnancy BMI (ρ=0.80, p<0.01). We also showed that among women delivering preterm, a 1 kg/m^2 increase in pre-pregnancy BMI predicted a 2% increase in breastmilk LA, suggesting that those with a pre-pregnancy BMI >30 kg/m^2 compared to a normal BMI produce breastmilk with 20% higher LA.[111] These findings are noteworthy given higher LA in milk from WLHIV in SA (mean=23.6 mol%) vs. WLHIV in Tanzania (mean=12.3 mol%) and Canadian women without HIV (mean=13.6 mol%).[112-114] While WLHIV breastfeed commonly,[115] no data exist on breastmilk FA, DTG exposure, and child metabolic health.

Table 3. Lipids at 12m by *in utero* HIV/ARV exposure

Lipid fraction, mg/dL	*HEU infants (n=57)*	*HUU infants (n=21)*	*p*
TC	159 (140-179)	139 (122-158)	0.02
LDL	90 (80-113)	77 (68-90)	0.03
HDL	43 (35-51)	35 (32-46)	0.06
TG	96 (75-140)	97 (62-122)	0.72

C. METHODOLOGY

Overall design. We will conduct a <u>prospective cohort study</u> following pregnant women from T1 to 24m PP along with their children (the <u>Main Cohort</u>, MC) to address Aims 1, 2, 2a, 3, and 3a, with substudies sampled from the MC to interrogate aims, including <u>nested prospective cohorts</u> (Aims 1a,) and <u>case-cohort studies</u> (Aims 1b,2b,3b,3c) (Table 4).

Setting. Research will take place at the Gugulethu and Mitchells Plain Community Health Centers (CHCs) in Cape Town. These are community-based facilities providing comprehensive primary care to two of the largest former township communities of Cape Town. At each CHC, research will be based at Midwife-Obstetric Units (MOU) which provide maternal and child health services with comprehensive antenatal and PP care with integrated HIV/ART. MTCT levels are 2-3% through 18 months of age. Breastfeeding is widespread (>80%) regardless of maternal HIV with a median duration of 6 months (IQR, 4-9m).

Table 4. Summary of study designs, comparison groups and key measures for analyses to address each of the Specific Aims

	Aim & Study (population)	*Design and Comparisons*	*Key measures for analysis*	*Sample size for analysis*
1	**Main Cohort** (*mothers*)	Prospective cohort study of all eligible pregnant women, comparing (i) HIV+ vs HIV- and (ii) HIV+ continuing DTG (cDTG) vs initiating DTG (iDTG) in pregnancy	Longitudinal changes in weight, adipose tissue mass (ADP)	n=1800
1a	**Nested cohort 1a** (*mothers*)	Prospective cohort study sampled from Main Cohort, comparing (i) HIV+ vs HIV- and (ii) cDTG vs iDTG	Maternal REE & Caloric intake	n=1240
1b	**Case-cohort study 1b** (*mothers*)	Case-cohort sampled from within nested cohort 1a<br>2 <u>case definitions</u>: women in the top deciles of the cohort in (i) adipose accrual by ADP between T1 and T3, & (ii) GWG in pregnancy<br><u>Comparator subcohort</u>: random sample of all women in MC<br>Comparisons by HIV+ vs HIV-; cDTG vs iDTG; and case vs comparator subcohort	Markers of systemic & adipose inflammation, gut integrity, satiety/hunger	n=840
2	**Main Cohort** (*mothers*)	Prospective cohort study of all eligible pregnant women, comparing (i) HIV+ vs HIV- and (ii) cDTG vs iDTG	*Metabolic health PP*: PP weight retention; adiposity (ADP); Dysglycemia (OGTT); IR (DIo, Mari, Matsuda); Dyslipidemia	n=1500
2a	**Main Cohort** (*mothers*)	Prospective cohort study of all eligible pregnant women, comparing (i) HIV+ vs HIV- and (ii) cDTG vs iDTG	Longitudinal changes in weight, adipose tissue mass (ADP), Metabolic health PP (see above)	n=1500
2b	**Case-cohort study 2b** (*mothers*)	Case-cohort sampled from within MC<br>2 <u>case definitions</u>: women with (i) poorest decile of DIo values at 24m PP, & (ii) women in top decile of PP weight retention in cohort at 24m postpartum<br><u>Comparator subcohort</u>: random sample of all women in MC<br>Comparisons by HIV+ vs HIV-; cDTG vs iDTG; and case vs comparator subcohort	Maternal metabolites, lipid subspecies, eicosanoids	n=960
3	**Main Cohort** (*infants/children*)	Prospective cohort study of all eligible children, comparing (i) HIV-exposed vs HIV-unexposed and (ii) born to HIV+ mothers cDTG vs iDTG	*Neonatal metabolic health*: Weight; Adipose tissue mass (ADP, skinfold thickness); IR (Cord blood glucose, insulin, C-peptide); *Child metabolic health*: Weight; Adipose tissue mass (skinfold thickness); IR (HOMA-IR); Dyslipidemia	n=1500
3a	**Main Cohort** (*infants/children*)	Prospective cohort study of all eligible children, comparing (i) HIV-exposed vs HIV-unexposed and (ii) born to HIV+ mothers cDTG vs iDTG	Neonatal adipose tissue mass (ADP)<br>Neonatal metabolic health, child metabolic health (above)	n=1500
3b	**Case-cohort study 3b** (*infants/children*)	Case-cohort sampled from children with cord blood specimens within MC<br>3 <u>case definitions</u>: top deciles of: (i) neonatal adipose tissue mass from ADP; (ii) adipose tissue mass from skinfold thicknesses at 24m & (iii) LDL at 24m<br><u>Comparator subcohort</u>: random sample of all children in MC<br>Comparisons by HIV+ vs HIV-; cDTG vs iDTG; and case vs comparator subcohort	Cord blood insulin/C-peptide, metabolites, lipid subspecies, eicosanoids	n=750
3c	**Case-cohort study 3c** (*infants/children*)	Case-cohort sampled from children in Case-cohort study 3b<br><u>Case definitions</u> as for Case-cohort study 3b<br><u>Comparator subcohort</u>: random sample of all children in MC<br>Comparisons by HIV+ vs HIV-; cDTG vs iDTG; and case vs comparator subcohort	Breastmilk fatty acid profiles, breastmilk & infant blood DTG levels	n=250

Study Population. We will recruit up to n=1900 pregnant women and follow them to delivery and then as mother-infant pairs through 2 years PP

We will recruit women at their 1st ANC visit (T1). Enrollment will be distributed across three main exposure categories by HIV status and DTG use (hereafter, HIV/DTG exposure category):

(i) <u>WLHIV initiating DTG-based ART in pregnancy at the 1st ANC visit</u> (**iDTG**);
(ii) <u>WLHIV already on TLD prior to enrolment and continuing DTG through pregnancy</u> (**cDTG**); and
(iii) <u>women without HIV infection</u> (**HIV-**).

Inclusion and exclusion criteria. We will use the following eligibility criteria for all aims.
Inclusion criteria for all women:

- Confirmed pregnancy based on urine pregnancy test with viable gestation ≤ 18 weeks and 6 days by ultrasound
- Age 16 years or older
- No stated intention to relocate permanently outside of Cape Town through 2 years postpartum

For Women Living with HIV (WLHIV):

- Confirmed HIV infection based on medical record review and/or HIV antibody testing during antenatal care

For WLHIV continuing DTG in pregnancy (cDTG):

- Confirmed use of tenofovir 300mg + lamivudine 300mg/emtricitabine 200mg + dolutegravir 50mg (TLD) on the day of assessment

For WLHIV initiating DTG in pregnancy (iDTG):

- Planned initiation of TLD on the day of assessment or within 1 week thereafter, including women switching from efavirenz-based regimen

For women without HIV (HIV-):

- Confirmed HIV status by HIV antibody testing during antenatal care
    - Note that HIV- women will be assessed with ongoing HIV testing at select study visits to detect incident HIV infection; any woman seroconverting during the follow-up period will be censored.

Additional inclusion criteria for Aim 3, 3a, 3b, 3c:

- Live birth with no severe congenital anomalies

Additional inclusion criteria for Aim 3c:

- Infant breastfed after 1 week of age
- Infant alive at 6 weeks of age (time of primary breastmilk sample collection)

Exclusion criteria for all women:

- In the opinion of the investigator, unable to provide informed consent due to mental or physical condition
- In the opinion of the investigator, unable to undertake BodPod assessment due to mental (eg, active psychosis or severe claustrophobia) or physical condition (eg, weight >250 kg).
- Currently being treated for any form of diabetes mellitus or hypertensive disorder based on participant self-report and medical record review

Rationale for Involving Proposed Populations. This study involves pregnant women living with HIV. This is because our study focuses on the impact of DTG on metabolic health of women and children in pregnancy, delivery and beyond. In order to understand the potential adverse effects of DTG, women will be ≤18 weeks and 6 days gestation at enrollment to allow for detection of metabolic abnormalities as they begin to develop during the course of pregnancy. All participants who screen positive for gestational diabetes, metabolic syndrome, or hypertension will be referred for specialized maternity care, in accordance with South African guidelines. Because we will be using DOH public clinics as our study sites and recruiting women directly from these clinics, all participants will be under the supervision of a physician and all participants with HIV will be prescribed ART. Study staff will NOT make changes to clinical care or ART regimens and participants will be encouraged to speak with their doctor if they have concerns about their medications or side effects of these medications or if they have concerns about their diagnosis of gestational diabetes or a hypertensive disorder. Any unexpected ethical issues that arise with our participants will be discussed immediately with the PI. All participants will receive a resource packet including information on pregnancy and PMTCT as well as references to local health and social services. (Please also see 'Additional Protection for Pregnant Women' section on page 26 below)

Recruitment. Women will be recruited during their first ANC visit by a research counsellor. Following our standard practice in this setting, we will work with nurse-midwives providing routine ANC in each MOU to help identify potentially eligible patients, with research staff coordinating closely with MOU staff. A short screening tool will be used to assess eligibility and women's potential interest in participation, with a screening log used to document reasons for any woman not participating in the study. Eligibility questions will be embedded within a set of foil questions so that ineligible individuals will not be able to guess the true purpose of the study from the eligibility criteria. We will attempt to enroll a random sample of women seeking antenatal care, stratified by HIV and ART initiation timing status. Eligible and interested women will then undergo an informed consent process, where a research

counsellor will explain the study procedures in a private setting and provide forms in English, Afrikaans, and isiXhosa.

Informed Consent and enrolment. All informed consent procedures will follow protocol approved by the University of Cape Town HREC, Columbia University and Ann & Robert H. Lurie Children's Hospital of Chicago/Northwestern University IRBs. Prior to enrolment the informed consent process will be delivered in participants' home language (isiXhosa, Afrikaans, or English) by a trained fieldworker following a standardized script. This script will detail the purpose of the study, all study visits and procedures for the woman and child, as well as the risks and benefits that they may encounter during the study. Here and throughout it will be emphasized to participants that:

- Participation is entirely voluntary, and their choice regarding participation will in no way influence the quality of antenatal or postpartum routine medical care received;
- Women are allowed to ask questions - the staff member will probe for complete understanding during key points of the consent form;
- Women are allowed to take as much time as they need to make a decision; and that
- Women may exit the study at any time for any reason without compromising the quality of health care received.

In addition, informed consent will contain separate sections allowing women to opt in/out for specific study activities including, but not limited to, specimen storage/potential future use and testing of genetic material as well as sharing of the participant's de-identified data on the NICHD Data and Specimen Hub (DASH).

Women will be asked to complete a second informed consent process and signed form at the first postnatal visit agreeing to participation of their child in the study and confirming willingness to continue, with their child, in the study through 24 months.

Signed documentation of informed consent will be taken from all participants. Informed consent documents will be translated into isiXhosa and Afrikaans, and back-translated to ensure accuracy. Following IRB and HREC guidelines, women who are illiterate and unable to provide their signature will be asked to provide a thumbprint, which will be independently witnessed.

All participants will be provided the contact numbers of the UCT Principal Investigator Prof Landon Myer and UCT HREC chair Prof Marc Blockman to answer questions that the participant might have about the study or one's rights as a human subject. Completed, signed consent forms will be kept in a locked filing cabinet in our secured research office. The participant will be allowed to take a copy of the consent form home to have as a reference but will also be reminded that someone finding the form may result in a breach of confidentiality.

Language. The trained research staff will be trilingual in English, isiXhosa and Afrikaans, and study activities will be conducted in the participant's language of preference. Study questionnaires will be written in English, translated to isiXhosa and Afrikaans by a professional translator and back translated to English to ensure consistency in questions between the languages. Co-Investigators and clinic staff will be consulted to ensure that content meanings are consistent between the two languages and account for cultural terminology and understanding. We draw from established research networks at University of Cape Town to hire RAs experienced in clinical research and aware of the linguistic precision needed for this work.

Data Collection. All women will complete informed consent procedures with a trained research assistant prior to data collection. Data will be collected via face-to-face interviews and physical assessments during <u>10 study visits</u> separate from routine clinic visits, these

visits include 3 antenatal (≤18, 24-28 and 32-36 weeks) and 7 postnatal visits (<2, 6, 12, 24, 48, 72 and 96 weeks). Data collection procedures and schedule are summarized in Table 5. Data will be collected by trained data collectors. All instruments including those for measuring anthropometry, blood pressure, body composition and resting energy expenditure will be calibrated and maintained regularly following manufacturer's instructions. Additional data on maternal health during pregnancy and birth outcomes will be abstracted from clinical records.

Specimens (whole blood) will be transported to the South Africa National Health Laboratory Service (SA-NHLS) for real-time analysis of dysglycemia and IR. Other specimens (whole blood, cord blood, placenta, urine and breastmilk) will be transported to UCT Institute of Infectious Disease and Molecular Medicine (IDM) laboratories for processing (to obtain plasma, buffy coat and serum) or crude storage at -80℃. Some of the stored specimens will later be analysed for markers of systemic and adipose inflammation at UCT and some will be shipped to the Albert Einstein Metabolomics Core for analysis of metabolites, lipid subspecies and eicosanoids.

Study procedures. We will conduct the following procedures for the **10 study visits**:

○ **Visit 1: ≤18 weeks' gestation (enrolment)**

Gestational age and fetal growth. Obstetric ultrasound will be used for pregnancy dating and to monitor intrauterine growth using standard fetal biometry.[132] With minimal but appropriate technical modifications, the ultrasound will be used to assess maternal *hepatic steatosis* using scored B-mode imaging operated by a trained research ultrasonographer.[187-189] An experienced radiologist will interpret ultrasound imaging for hepatic steatosis, blinded to participant information and measurement timing. At Visit 1- ultrasound results (including estimated date of delivery, findings of clinical significance) will be shared with the public sector clinical services for inclusion in routine medical record.

Locally-established questionnaires will be employed to measure *demographics and health status* including household composition[165,166]; health care utilization, alcohol (AUDIT), tobacco and drug use (DUDIT), *anxiety (GAD-7) and depression (EPDS), COVID-19, physical activity (IPAQ, PPAQ), interpersonal violence, satiety/appetite (TFEQ), food insecurity (PDIS), gastro-intestinal tract symptoms and unintended pregnancy (LMPU)*. *Maternal HIV disease and ART use* will include history of HIV-related conditions while ART use will include previous ART and duration of current ART, as appropriate. *ART adherence* will be measured via self-report.

Caloric intake will be measured via 7-day and 24-hour recall using the Western Cape Food Frequency Questionnaire (WC-FFQ), a locally-developed instrument whose validation included data from the Gugulethu and Mitchells Plain communities.[220] Using the SA Medical Research Council's Foods Composition Database,[221] we will convert WC-FFQ responses to estimate energy, carbohydrate, protein, fat, and fiber intake based on 1741 commonly consumed foods in SA.

Anthropometry will serve as secondary measures of adiposity using validated equations[148,150,151] for women and children. We have extensive experience measuring adiposity in pregnant women[35,144,151-153] and children[40,148] using these methods. Anthropometry will be measured using our prior SOPs for quality control (QC), including duplicate measures and re-training. Maternal anthropometry will include weight and height (for BMI), middle-upper-arm circumference, and skinfold thicknesses with calipers (triceps, subscapular, and suprailiac).

Adipose tissue mass will be measured by air displacement plethysmography (ADP),[139] providing rapid, reliable, non-invasive measures using the BodPod system (Cosmed, Rome, Italy). This system uses whole-body densitometry to determine fat and fat-free mass in an

assessment lasting 3-5 minutes. ADP has been widely validated in adults, including the context of HIV infection and pregnancy, with excellent acceptability and accuracy.[35,140-146] Per our prior research established hydration constants specific to late pregnancy will be used, and thoracic volume will be measured.[35,144,146]

Resting energy expenditure will be measured via indirect calorimetry, performed in selected subjects at T1 and T3 using the Q-NRG system (CosMed, Rome, Italy) a widely used research measure for REE validated across a range of BMI and disease states.[218,219] This measures respiratory volumes, oxygen consumption, and CO_2 production at 30s intervals to calculate REE using the abbreviated Weir equation and a fixed respiratory quotient. Assessments will be conducted after overnight fasting in dedicated research rooms while participants watch television or read; after a 20min period of initial quiet rest, participants will wear a canopy hood for another 20min while resting.

Dysglycemia and IR will be assessed using a 75g oral glucose tolerance test (OGTT) with insulin, glucose and C-peptide measurements collected at 0, 30, 60, and 120 minutes post OGTT. We will first measure 1st phase insulin secretion and clearance and β-cell function using the *Mari model*.[155-157] Next, we will assess insulin sensitivity using the *Matsuda Index*[158] which we have validated in pregnancy.[159] Lastly, we will use these measurements to calculate the *Oral Disposition Index (DIo)* which provides an accurate measure of insulin secretion relative to the amount of insulin sensitivity; DIo values of <1.24 have been shown to predict future DM risk.[160] Dysglycemia will be defined as impaired fasting glucose or impaired glucose tolerance.[161] Gestational diabetes (GDM) will be defined as meeting any one of the following glucose (mg/dL) criteria: fasting ≥92, 1h ≥180, or 2h ≥153.

Dyslipidemia will be assessed using fasting lipid profile [TC, high density lipoprotein (HDL), LDL, triglycerides (TG)].

We will also collect specimens for testing *HIV disease* progression (in WLHIV) and *HIV testing* (in women without HIV). Urine will be collected for testing of *glucose and protein excretions*. *Stored specimens* including plasma, serum, buffy coat in RNA later and urine will also be included. We will draw approximately 65 mL of blood and 10mL of urine for the assays and specimens listed above at this visit.

○ **Visit 2: 24-28 weeks' gestation**

We will measure *health status* including health care utilization, *COVID-19, caloric intake, satiety/appetite (TFEQ), socioeconomic status, HIV, obesity stigma and body image perception, green spaces (locator form), sleep quality (Berlin & Pittsburgh) and adverse childhood experiences*, *HIV disease and ART use*, *intercurrent medical history including concomitant medication use*, *ART adherence*, *community violence and social support, anthropometry* including weight, middle-upper-arm circumference and skinfold thicknesses with calipers (triceps, subscapular, and suprailiac) and collect specimens for testing *HIV disease* progression (in WLHIV) and *HIV testing* (in women without HIV). Urine will be collected for testing of *glucose and protein excretions*. *Stored specimens* including plasma, serum, buffy coat in RNA later and urine will also be included. We will draw approximately 25 mL of blood and 10 mL urine for the assays and specimens listed above at this visit.

- **Visit 3: 32-36 weeks' gestation**

Gestational age and fetal growth. Obstetric ultrasound will be used to monitor intrauterine growth using standard fetal biometry. Ultrasound results will only be shared with the public sector clinical services for inclusion in the routine medical record if there are findings of clinical significance that require further management.

We will measure *hepatic steatosis, health status* including health care utilization, alcohol (AUDIT), tobacco (ASSIST) and drug use (DUDIT), *anxiety (GAD-7) and depression (EPDS), COVID-19, physical activity (IPAQ, PPAQ), interpersonal violence, food insecurity (PDIS), household composition, HIV disease and ART use*, *intercurrent medical history including concomitant medication use*, *ART adherence*, *caloric intake, gastro-intestinal tract symptoms, anthropometry* including weight, middle-upper-arm circumference and skinfold thicknesses with calipers (triceps, subscapular, and suprailiac), *adipose tissue mass*, *resting energy expenditure, dysglycemia, IR*, *dyslipidemia* and collect specimens for testing *HIV disease* progression (in WLHIV) and *HIV testing* (in women without HIV). Urine will be collected for testing of *glucose and protein excretions*. *Stored specimens* including plasma, serum, buffy coat in RNA later and urine will also be included. We will draw approximately 65 mL of blood and 10mL of urine for the assays and specimens listed above at this visit.

- **Delivery**

We will collect *cord blood and placenta* (when available) from delivery, for use in relevant substudies (see laboratory measures below) and towards a repository of specimens for future research. We will collect up to 20 mL of cord blood at this visit.

- **Visit 4: < 2 weeks PP**
  - ***Mother***

We will measure *health status* including health care utilization, *COVID-19, HIV disease and ART use*, *intercurrent medical history including concomitant medication use*, *ART adherence, caloric intake, anthropometry* including weight, middle-upper-arm circumference, waist circumference, waist:hip ratio and skinfold thicknesses with calipers (triceps, subscapular, and suprailiac).

  - ***Child***

Adapted tools will be employed for the assessment of *birth defects, breastfeeding status and intercurrent medical history including concomitant medication use.*

Anthropometry will include assessment of weight, length, head and waist circumferences, and skinfold thicknesses with calipers (triceps, subscapular, suprailiac) for adipose estimation.

Adipose tissue mass will be measured by air displacement plethysmography (ADP),[139] providing rapid, reliable, non-invasive measures using the PeaPod system (Cosmed, Rome, Italy). This system uses whole-body densitometry to determine fat and fat-free mass in an assessment lasting 3-4 minutes. We have previously validated ADP from one week to 6 months of age or 8kg, and for both premature and term infants.[147-149]

- **Visit 5: 6 weeks PP**
  - ***Mother***

We will assess *birth defects* and *health status* including health care utilization, *COVID-19, HIV disease and ART use*, *intercurrent medical history including concomitant medication use*, *ART adherence, sleep quality (Berlin & Pittsburgh), anthropometry* including weight, middle-upper-arm circumference, waist circumference, waist:hip ratio and skinfold thicknesses with calipers (triceps, subscapular, and suprailiac) and collect specimens for testing *HIV disease* progression (in WLHIV) and *HIV testing* (in women without HIV). Urine will be collected for testing of *glucose and protein excretions*. *Stored specimens* including plasma, serum, buffy coat in RNA later, urine and breastmilk will also be included. We will collect approximately 25 mL of blood for the assays and specimens listed above, 10 mL of urine and 20 mL of breastmilk for this visit.

  - ***Child***

We will examine *breastfeeding status and intercurrent medical history including concomitant medication use, anthropometry* including weight, length, head and waist circumferences, and skinfold thicknesses with calipers (triceps, subscapular, suprailiac) for adipose estimation, and will collect *stored specimens* including plasma, serum and buffy coat in RNA later. We will collect approximately 8 mL of blood for the specimens at this visit.

- **Visit 6: 12 weeks PP**
  - ***Mother***

We will measure *health status* including health care utilization, *anxiety (GAD-7) and depression (EPDS), COVID-19, physical activity (IPAQ, PPAQ), food insecurity (PDIS), caloric intake (24-hour recall), gastro-intestinal tract symptoms, HIV disease and ART use*, *intercurrent medical history including concomitant medication use*, *ART adherence, anthropometry* including weight, middle-upper-arm circumference, waist circumference, waist:hip ratio and skinfold thicknesses with calipers (triceps, subscapular, and suprailiac) and collect specimens for testing *HIV disease* progression (in WLHIV) and *HIV testing* (in women without HIV). *Stored specimens* including plasma, serum, buffy coat in RNA later, breastmilk will also be included. We will collect approximately 25 mL of blood for assays and specimens listed above and 20 mL of breastmilk at this visit.

  - ***Child***

We will examine *breastfeeding status and intercurrent medical history including concomitant medication use, caloric intake, child care, anthropometry* including weight, length, head and waist circumferences, and skinfold thicknesses with calipers (triceps, subscapular, suprailiac) and *adipose tissue mass*.

- **Visit 7 & 9: 24 and 72 weeks PP**
  - ***Mother***

We will measure *health status* including health care utilization, alcohol (AUDIT), tobacco (ASSIST) and drug use (DUDIT), *COVID-19, interpersonal violence, green spaces (locator form), household composition, socioeconomic status, obesity stigma and body image perception, sleep quality (Berlin & Pittsburgh), HIV disease and ART use*, *intercurrent medical history including concomitant medication use*, *ART adherence, caloric intake, community violence, social support and menstruation (Visit 9 only), anthropometry* including weight, middle-upper-arm circumference, waist circumference, waist:hip ratio and skinfold thicknesses with calipers (triceps, subscapular, and suprailiac) and collect specimens for testing *HIV disease* progression (in WLHIV) and *HIV testing* (in women without HIV). *Stored specimens* will include plasma, serum and buffy coat in RNA later. We will collect approximately 25 mL of blood for the assays and specimens listed above at these visits.

  - ***Child***

We will examine *breastfeeding status and intercurrent medical history including concomitant medication use, anthropometry* including weight, length, head, and waist circumferences, and skinfold thicknesses with calipers (triceps, subscapular, suprailiac).

- **Visit 8 & 10: 48 and 96 weeks PP**
  - ***Mother***

We will measure *health status* including health care utilization, alcohol (AUDIT), tobacco and drug use (DUDIT*), anxiety (GAD-7) and depression (EPDS), COVID-19, physical activity (IPAQ, PPAQ), caloric intake (24-hour recall), gastro-intestinal tract symptoms, satiety/appetite (TFEQ), food insecurity (PDIS)*, *HIV disease and ART use*, *intercurrent medical history including concomitant medication use*, *ART adherence, caloric intake, anthropometry* including weight, middle-upper-arm circumference, waist circumference, waist:hip ratio and skinfold thicknesses with calipers (triceps, subscapular, and suprailiac)

a*dipose tissue mass*, r*esting energy expenditure, dysglycemia, IR*, d*yslipidemia;* and collect specimens for testing *HIV disease* progression (in WLHIV) and *HIV testing* (in women without HIV). Urine will be collected for testing of *glucose and protein excretions*. *Stored specimens* including plasma, serum, buffy coat in RNA later and urine will also be included. We will collect approximately 65 mL of blood for the assays and specimens listed above at these visits and 10 mL of urine at this visit.

- ***Child***

We will examine *breastfeeding status and intercurrent medical history including concomitant medication use*, *anthropometry* including weight, length, head and waist circumferences, and skinfold thicknesses with calipers (triceps, subscapular, suprailiac), *adipose tissue mass*. In addition, we will assess *dysglycemia and IR* using pre-prandial insulin and glucose (for HOMA-IR estimation) and *dyslipidemia* using pre-prandial lipids [TC, high density lipoprotein (HDL), LDL, triglycerides (TG)]. We will also collect *stored specimens* including plasma, serum and buffy coat in RNA later. We will collect approximately 15 mL of blood for the assays and specimens listed above at these visits.

- **Medical Record abstraction**

We have extensive experience in utilizing data from routinely collected electronic medical records in this setting. Data will be requested from the Western Cape Provincial Data Centre using systems and procedures developed as part of MCH-ART (451/2012), and we will use participants' provincial folder number (requested as part of tracing information) to facilitate an accurate electronic data request. Permission to review electronic clinical records of participants will be included in informed consent documents. Following standard procedures, we will request approval from HREC at UCT and from the Provincial Department of Health to access electronic medical records from the Provincial Health Data Centre (PHDC). Established procedures are in place to ensure patient confidentiality. Identifying information of enrolled participants may be shared with the PHDC as encrypted and password-protected files sent via the UCT secure file sender. Access to PHDC data will be requested for ORCHID study, with access limited to participants enrolled in the study. The Site Coordinator will link these data to participants' unique participant identifiers. Once all routine data are linked the folder number will be removed and only the study identification number will be used. Data abstraction will be conducted by trained fieldworkers working under the close supervision of the Site Coordinator.

- **Women**: Data to be abstracted will include *HIV testing, maternal HIV history, ART use, HIV RNA viral load, CD4+ number and percent, past and current obstetric history, intercurrent medical history including medications, hospitalizations, concomitant medications, family planning including contraception*. *ART adherence* will be obtained from pharmacy records using approaches we have validated previously in this setting.[175-178]

- **Children**: Data to be abstracted will include *delivery information and birth outomes* [birthweights, gestational age (GA), congenital anomalies,[180] etc.], *vaccination history, HIV testing, intercurrent medical history including ambulatory presentations (such as respiratory or diarrheal illnesses), medications, hospitalizations*.

- **Laboratory measures**
  - ***Real-time analysis***

Aim 2. *H2: DTG use during pregnancy and postpartum will be associated with adverse maternal metabolic health (dysglycemia, IR, and dyslipidemia).*

Aim 3: *H3: In utero DTG-exposure will be associated with adverse neonatal and child metabolic health (insulin resistance, dyslipidemia).*

Real time analysis of blood specimens will be conducted at SA-NHLS in Groote Schuur Hospital in Cape Town. Specimens for study-specific HIV disease progression (in WLHIV) will be drawn at each study visit and batch-tested (Abbott RealTime HIV-1) as a marker of HIV treatment effects in the cDTG and iDTG groups. The tests that will be conducted for this include *HIV viral load* and *CD4 count* cell count. We will estimate cumulative viremic burden over pregnancy[171] and adjust for this as viremia is known to be associated with inflammation, obesity and IR.[172-174] In addition, we will conduct *HIV antibody* testing in women without HIV at each study visit to detect incident HIV infections.[179] Tests for dysglycemia and IR will include analysis of *glucose, insulin and c-peptide*, while tests for dyslipidemia will include analysis of *TC, high density lipoprotein (HDL), LDL and triglycerides (TG).*

- ***Storage at UCT for later analysis***

Aim 1b: *H1b: DTG use will be associated with decreases in adiponectin and increases in sCD163, sCD14, leptin, IL-6 (adipose inflammation), I-FABP (gut integrity), as well as α-MSH and ghrelin (satiety/hunger).*
This analysis will be performed at the IDM at UCT (www.idm.uct.ac.za) where Prof Myer is a PI and maintains laboratory facilities and close collaborations with chemical pathology, immunology and genetics laboratories.

Design / Sampling. Two case groups are proposed based on the findings of Aim 1: (i) women in the top decile in the cohort of adipose accrual between T3 and T1 (n=180); and (ii) women in the top decile in the cohort of GWG during pregnancy (n=180; we anticipate 33% overlap between these case definitions for estimating n=300 cases across both definitions). A single comparator subcohort will be sampled at random independently of HIV/DTG category from the baseline MC population at a 3:1 ratio (n=540 comparators total).

Measures. Using stored plasma from T1 and T3, we will test on cases and comparators markers of systemic and adipose inflammation (*sCD163, sCD14, IL-6, leptin, adiponectin*), gut integrity (*I-FABP*), and satiety/hunger (*α-MSH, ghrelin*) using ELISAs (Abcam, Cambridge, MA) at the University of Cape Town.

- ***Storage at UCT for later shipping to USA***

This analysis will be performed at the Albert Einstein Metabolomics Core at the Albert Einstein College of Medicine in New York, United States.

Aim 2b: *H2b: A signature cluster of lower TAG and LPC PUFAs in pregnancy will be potentially beneficial in pregnancy but associated with progressive adverse metabolic health PP.*

Design / Sampling. Two case groups defined at 24m are proposed based on the findings of Aim 2: (i) the poorest decile of DIo values in the cohort (n=200);[233] and (ii) women in the top decile of PP weight retention (PPWR) (n=200; we anticipate 20% overlap between these case definitions for an anticipated total of 360 cases across both definitions). A single comparator subcohort will be sampled at random from the baseline MC population, at a 3:1 ratio, sampled independently of HIV/DTG exposure (n=600 total).

Measures. Metabolomic profiling will use ~1.0 mL of plasma from each participant collected at T1 and T3 and stored at -80ºC before shipping to the Albert Einstein Metabolomics Core. We will use LCMS/MS developed for the Sciex 6500+ QTRAP system (S10 Award, Kurland) which provides comprehensive data on approximately 600+ small metabolites, 1300 lipid species in 26 lipid classes, and ~100 distinct eicosanoids.

Aim 3b: *H3b: A fetal cord blood signature of lower phospholipids containing PUFAs as well as higher pro-inflammatory eicosanoids derived from arachidonic acid (an n-6 PUFA) and lower anti-inflammatory eicosanoids derived from EPA or DHA (n-3 PUFAs) will be associated with adverse child metabolic health.*

Design / Sampling. Sampling will be restricted to infants with cord blood specimens available, estimated to be >60% of the MC. Three case groups defined at different ages are proposed based on the findings of Aim 3: (i) children in the top decile of fat mass in the MC

according to neonatal ADP (from Aim 3; n=150), (ii) children in the top decile of adipose tissue mass in the cohort at 24 months based on sum of skinfolds[148] (n=150) and (iii) children in the top decile of LDL values in the cohort at 24 months (n=150; we anticipate 33% overlap between these three case definitions leading to an anticipated 300 cases in total). A single comparator subcohort will be sampled at random from children in the MC cord blood specimens available, at a 3:1 ratio per case and sampled independently of HIV/DTG exposure category (n=450 total). To facilitate secondary inferences we will preferentially sample comparators by maternal inclusion in Case-cohort study 2b.

Measures. Cord blood will be assayed for glucose, insulin and C-peptide. In addition, metabolomic profiling of cord blood specimens will parallel the measures described for case-cohort study 2b, above. We have an established track record of collecting delivery specimens from previous NIH-funded research (PIMS 739/2014). Using our established SOPs we anticipate conservatively collecting >60% of delivery specimens. Here, two 3 mL plasma cord blood specimens will be collected from placental vessels within 5 minutes of delivery with storage at -80°C within 12 hours of delivery.

Aim 3c: *<u>H3c</u>: High n-6: n-3 (omega-6: omega-3) FA ratio, PUFA distribution among complex lipids, and DTG exposure in breastmilk will be associated with adverse child metabolic health.*

Design / Sampling. This analysis will use a case-cohort design, randomly sampling those children with maternal breastmilk specimens available from Case-Cohort study 3b (above). A total of 250 children will be included: 50 from each of the three case groups (n=100 presuming 33% overlap between case definitions, leading to an anticipated 100 cases across definitions) and 150 for the comparator subcohort.

Measures. Metabolomic profiling of breastmilk specimens will parallel the measures described for case-cohort study 2b (above), comprehensively identifying fatty acids and their distribution among lipid species. We have extensive experience collecting breastmilk measures in this setting with high levels of acceptability. DTG levels will be measured in both breastmilk and infant plasma using LC-MS.[29,251]

- ***Blood volumes***

Blood volumes are an important consideration for this protocol and will be minimized to the greatest extent possible at every visit. (See also Section E. Potential Risks) We will follow NIH Clinical Center guidance for maximal allowed blood volumes allowed in research. Infant blood will be collected by trained pediatric nurses. At the 48wk and 96wk visits approximately 15 mL will be drawn at each visit which will yield adequate amounts for planned assays as well as additional aliquots to be stored in the event preliminary data suggest the need for additional testing. The NIH recommends a limit of 3 mL/kg per single blood draw and a limit of 7 mL/kg in any 8-week period. The 3rd percentile and 97th percentile weight for males at 48 wks is 7.8 kg and 12 kg respectively, making the range of acceptable blood volume draw at this visit between 23.4 mL and 36 mL. At 96 wks of age the 3rd percentile and 97th percentile weight for males is 10.5 kg and 15.5 kg, making the range of acceptable blood volume draw at this visit between 31.5 mL and 46.5 mL. Therefore, we are well within the NIH guidance for maximum blood volume during phlebotomy of children. For pregnant and non-pregnant women, we will not exceed 65 mL of blood drawn at any visit in a 24 hour period which is well under 3% of the total blood volume of a 50 kg individual.

All procedures for data collection (regardless of source) will be outlined in a study manual of procedures (MOP), with individual activities guided by standard operating procedures (SOP; e.g., questionnaire administration, anthropometry, blood pressure measurement, ADP, OGTT, USG, phlebotomy, specimen preparation and storage). We have a bank of well-developed SOPs based on the HREC-approved and NIH funded research (*PIMS* 739/2014, *MCH-ART* 451/2012, *BPOS* 541/2015, *IUD-CT* 283/2012, *PACART* UK MRC 194/2015, *PrepPP* 297/2018).

Table 5. Summary and schedule of key study measures

**Measure** (outcomes in **bold**)	**Source**	**Antenatal**				**Delivery**	**Postnatal**						
**Main Cohort: Maternal (Aims 1, 2, 2a)**		<18w (T1A)	<20w (T1B)	24-28w (T2)	32-34w (T3)	0d	<2w	6w	12w	24w	48w	72w	96w
Demographics & health status	Questionnaire, Clinical data abstraction incl obstetric/medical history	X											
HIV disease & ART use	Questionnaire, Clinical data abstraction, CD4,[1] HIV VL,[1] HIV testing[2]	X		X[3]	X[3]		X	X[3]	X[3]	X[3]	X	X[3]	X
Depression & anxiety	EPDS, GAD-7		X		X				X		X		X
Intercurrent medical history	Questionnaire, Clinical data abstraction including concomitant meds			X	X		X	X	X	X	X	X	X
COVID Questionnaire	Adapted iCAP covid survey	X		X	X		X	X	X	X	X	X	X
Physical activity	IPAQ, PPAQ		X		X				X		X		X
Family & household composition	Standard questionnaire in this setting		X		X					X		X	
Economic activity & socio-economic status	Standard questionnaire in this setting			X						X		X	
Smoking, alcohol & drug use	ASSIST, AUDIT & DUDIT	X			X					X	X	X	X
Interpersonal violence	WHO (IPV)				X					X		X	
Caloric intake	24-hour recall		X		X				X		X		X
Food intake	7-day recall indicator FFQ			X								X	
Satiety/appetite	TFEQ	X		X							X		X
HIV stigma	Kalichman			X								X	
Obesity stigma & body image perception	Fat phobia & WBIS-M, Stankard silloetes			X						X		X	
Food insecurity	PDIS	X			X				X		X		X
Unintended pregnancy	LMPU	X											
Sleep quality	Berlin + Pittsburgh			X				X		X		X	
Adverse childhood experiences (ACES)	Standard questionnaire in this setting			X									
Menstruation regularity	Standard questionnaire in this setting											X	
Green spaces	Locator form (Geomapping)			X								X	
Community violence	Life events checklist			X								X	
Social support	Social networks questionnaire			X								X	
Gastro-intestinal tract symptoms			X		X				X		X		X
Sexual identity		X											
**Anthropometry, blood pressure**	**Stadiometer, scale, calipers,** tapes, Sphygmomanometry	X	X	X	X		X	X	X	X	X	X	X
**Adipose tissue mass**	**Air displacement plethysmography**	X*	X*		X						X		X
**Dysglycemia, IR** (**DIo,** Matsuda)	**Oral glucose tolerance test**		X		X						X		X
Hepatic steatosis	Ultrasound		X		X								X
**Lipid profile**	**Fasting lipid profile**		X		X						X		X
Stored specimens for substudies	Maternal plasma/serum, whole blood, breastmilk, urine		X	X	X			X	X	X	X	X	X
**Measures for Main Cohort: Fetal/Child (Aims 3, 3a)**													
Gestational age, fetal growth	Obstetric ultrasound	X			X								
Delivery information	Clinical data abstraction					X							
Neonatal exam	Clinical data abstraction & surface exam form						X	X					
Intercurrent medical history	Questionnaire, Clinical data abstraction including concomitant meds						X	X	X	X	X	X	X
Breastfeeding status/history	Standard questionnaire in this setting						X	X	X	X	X	X	X
Caloric intake	24-hour recall								X		X		X
Child care	Standard questionnaire in this setting								X		X		X
Feeding practices	CFPQ												X

Neurodevelopment	WHO (Global scale for early development)										X		X
Infant fasting											X		X
**Anthropometry**	**Stadiometer, scale, calipers**					X	X	X	X	X	X	X	X
**Adipose tissue mass**	**Air displacement plethysmography, Skinfold thickness**						X		X		X		X
**IR**	**Pre-prandial insulin, C-peptide, & glucose (HOMA-IR)**										X		X
**Lipid profile**	**Pre-prandial lipid profile**										X		X
Stored specimens for substudies	Cord blood, infant plasma/serum, whole blood, placenta					X		X			X		X
**Measures for Nested Substudies**													
Maternal REE	Indirect calorimetry (Nested cohort 1a / Aim 1a)		X		X						X		X
Markers of systemic & adipose inflammation, gut integrity, satiety/hunger	Plasma markers of adipose inflammation (IL-6, sCD163, sCD14, leptin, adiponectin), gut integrity (I-FABP), and satiety/hunger (α-MSH, ghrelin) (Stored specimens in Case-cohort study 1b / Aim 1b)		X		X								
Maternal metabolites, lipid subspecies, eicosanoids	Metabolomics assays - Sciex Triple Quad 6500+ LC-MS/MS system (Stored Specimens: Case-Cohort study 2b / Aim 2b)		X		X								
Cord blood insulin/C-peptide, metabolites, lipids, eicosanoids	Metabolomics assays - Sciex Triple Quad 6500+ LC-MS/MS system (Stored Specimens in Case-Cohort study 3b / Aim 3b)					X							
Breastmilk FA profiles, Breastmilk & infant blood DTG levels	Metabolomics assays - Sciex Triple Quad 6500+ LC-MS/MS system (Stored Specimens in Case-Cohort study 3c / Aim 3c)							X	X				

[1] cDTG and iDTGc only; [2] Women without HIV only; [3] No CD4 testing
** Adipose tissue mass will be measured by air displacement plethysmography at either T1A or T1B, based on staff determination*

Retention procedures. Following all enrolment procedures, women will be provided with a study-specific appointment card with the dates of their next study visits and emergency contact information if needed. Study-specific follow-up visits will be conducted at our research facilities located on the grounds of the Gugulethu and Mitchells Plain MOUs and will be timed to coincide with routine ANC visits at 24-28 weeks (second trimester, T2) and 32-34 weeks gestation (third trimester, T3). Following our established practice, we work closely with MOU staff to coordinate follow-up visits schedules and minimize the burden on participants.

Delivery specimens. Our approach to collecting delivery specimens, including cord blood (for Aim 3b) and placentas (when available as an additional stored specimen) will follow practices developed for previous research (HREC 739/2014) where we have successfully obtained 60-70% of delivery specimens.

- The study's intention to collect delivery specimens is communicated to women at enrolment and each antenatal visit, and women are provided with a study appointment card that includes a 24-hour phone number with instructions to contact at the onset of labour.
- After their T3 visit, women are phoned weekly to check on their well-being and remind them about delivery procedures until 36 weeks gestation, then twice weekly to 39 weeks gestation, then every other day until they go into labour.

Tracing information will be collected at enrollment and updated at each participant contact. Information collected will include residential location (including alternate residences), cell phone information including alternate contact numbers (we have documented that individually-held cellphones are owned by >96% of women attending the Gugulethu MOU), social media handles, as well as the names and contact information of (i) a trusted neighbor and (ii) a friend or family member who does not live with the participant. All the tracing information we collect and our tracing activities are highly sensitive to issues of HIV disclosure and refer to women's participation in a "study about the health of women and babies" generically.

All tracing procedures are based on established practices employed for previous HREC-approved cohort research in this setting. At each site a dedicated team of staff is devoted to participant tracing, and retention will be monitored using a study-specific visit tracking database. Reminder calls will be made to participants 1 week, 3 days and 1 day before their study visits, and additional tracing including home visits, for women who do not attend the study visit on its scheduled date. The team will work with MOU staff to undertake follow-up phone calls on the date of a missed visit, the following day, and three days later. Home visits to trace participants who miss a study visit will be made by a dedicated tracing team with fieldworkers drawn from local communities.

Following our established practice, we will continue telephone contact with participants between in-person visits that are six months apart to maintain relationships and update tracing information (i.e. at 9, 15 and 21 months postpartum).

Compensation. All women participating in the study will be provided the equivalent of R150 (USD $11) for the ≤18w (T1A) visit which takes about 1.5 hours, R250 (USD $18) for the 6 study visits (24-28w, <2w, 6w, 12w, 24w and 72w) that take up to 3 hours; and R350 (USD $24) for the 4 study visits (≤18w (T1B), 32-34w, 48w and 96w) that take more than 3 hours to complete, for transport and missed opportunity costs for attending study-related visits (which are scheduled separately from routine health care visits). In addition, receipt of cord blood specimen will be incentivised an additional amount of R300/USD $21 at the <2w or 6w postnatal visits. Attendance of 12w to 96w postnatal visits will also be incentivised a total of R1000/USD $71 collectively at the last 96w visit. In case of missed visits, a total of the weighted amount for each visit will be provided. Weighted amount for each visit will be R50/USD $4 (12w), R50/USD $4 (24w), R200/USD $14 (48w), R50/USD $4 (72w) and

R650/USD $46 (96w). There will be no participant reimbursement for routine health care visits and no reimbursement to attend antenatal care or post-natal care services.

Identification of health care needs during study procedures. At every study measurement visit, any woman found to have an unmet health need (whether medical, obstetric, postpartum family planning, or related to mental health or substance use) will be referred to the relevant service within the Gugulethu or Mitchell's plain CHC or higher levels of care, as appropriate. In particular:

- Any women who screen positive for hypertension, metabolic syndrome, or gestational diabetes as part of the study will be referred to a medical provider for specialized care. Maternity care, including specialized maternity care, is available in South Africa at no cost. Therefore, we do not anticipate any additional financial burden on participants.
- Any woman found to have defaulted ART during the course of the study will be re-referred to the relevant ART service (general adult or MCH-focused) via the Gugulethu or Mitchell's plain MOU.
- Any woman found to be non-adherent to ART at any study measurement visit will receive intensive counselling on ART use and be referred to the relevant ART service for follow-up.
- Any woman found to be experiencing domestic or partner violence in any form will be referred to the main local NGO supporting victims of domestic violence (MOSAIC) and the South African Police Service (per the Domestic Violence Act, No. 116 of 1998) with follow-up to ensure adequate attention.
- Any child found to be HIV seropositive will be referred immediately to the nearest paediatric ART service for assessment and infant ART initiation.
- If any infant health care needs are identified, the infant will be referred to the appropriate paediatric health services via the Gugulethu or Mitchell's plain CHC.

Staff training. Prior to initiation of the study, all staff that will have contact with participants will take part in a multi-day study-specific training. The curriculum of the training will include: rationale, purpose, and scientific objectives of the study; study design and methodology; conduct of study assessments, tracking of participants, completion of study forms, and data collection; staff responsibilities; recruiting participants; procedures for enrolling participants into the study; universal precautions, communication skills, safety in the field, ethical guidelines for research including participants' rights; procedures for obtaining informed consent; and confidentiality requirements.

Study staff will receive a hands-on training that will include an introduction to data collection forms and procedures. Mock interviews will be an essential component of the training and protocol team members will act as both the trainer and the mock respondent. The trainer will take the staff through each step of the data collection process, from enrolling participants to ending the study visit and completing any necessary forms. Study staff will be given a chance to practice the English, isiXhosa and Afrikaans versions of all the assessments in order to discuss and resolve any issues. Training for study staff is expected to take approximately 2 weeks and additional follow-up training, as necessary.

Study staff members who will collect blood samples and conduct laboratory analyses will receive training in universal precautions, sample collection, and testing of study samples.

Expert technicians from Cosmed company in Italy will come to the study sites to train personnel that will be appointed to operate the BodPod and PeaPod systems.

In addition, staff will be trained in the management of crisis situations, including reports of abuse and domestic violence, that may be disclosed during study participation. The site will have established procedures for managing these situations and procedures in place for

providing counselling and appropriate referrals. Procedures for managing these issues will be outlined in study SOPs.

For all study staff, there will be additional training days scheduled during the study for refresher training. During these refresher trainings, study staff will review study procedures and discuss any challenges encountered. All staff who through the course of their work have knowledge of, or access to, personal information about participants will be required to complete training on patient confidentiality and sign a confidentiality agreement before the start of data collection.

D. DATA MANAGEMENT & ANALYSIS

Data collection and management: Following our established approaches data will be collected on tablets linked to a RedCap database developed for this study, with paper forms used for meta-documentation (e.g., specimen logs) as well as a backup in the event of technical failure. Externally derived data (such as from laboratory assays) will be imported using pre-specified templates. Weekly data merging and cleaning, including QC reporting will be led by data management staff at UCT with QC undertaken by data clerks at each site.

Sample size: The sample size for the MC is based on Aim 2, to test the association of HIV/DTG with postpartum metabolic health. We use the clinically-driven cutpoint of DIo<1.24 as this is strongly associated with increased risk of subsequent diabetes within 10 years.[160] We assume a 2-sided test at $\beta=0.1$ (90% power), a 1:2 ratio of HIV+ to HIV- women, and a background prevalence of DIo<1.24 of 2% at 2 years PP based on our prior research in this setting.[81] Further, we assume 87% cohort retention from T1 enrollment through 2 years PP, 6% participant withdrawal for medical reasons (including pregnancy losses, maternal/infant death, and <2% incident HIV in mothers or infants through 24m PP) and 2% censoring due to ART switches among WLHIV on DTG, all from previous studies.[179,190-194] Based on this, we estimate that n=1879 women (rounded up to n=1900) will be required to detect absolute increases in DIo<1.24 of 3-4% (to levels of 5-6% among WLHIV) for Aim 2; from this we estimate enrolling up to 1900 pregnant women into the study. When DIo is considered as a continuous variable, this will provide >90% power to detect mean DIo differences of 0.3 units, against a background value of 2.6 in women 25-34 years old in SA (SD=1.68).[81] In addition, based on our simulations for mediation analyses[195] this sample size will provide 77%-84% power for Aims 2a and 3a, and 87-91% power for Aims 1 and 3. In addition, this cohort size provides an adequate sampling frame for all substudies below.

Analysis. Data analyses will use R (R Foundation, Vienna, Austria). Throughout, descriptive and bivariable statistics will follow standard approaches; research site will be used as a variable for descriptive purposes, but we do not have *a priori* hypotheses regarding site differences and all hypotheses will be examined combining data from both sites. Person-time in prospective analyses will be included as long as women remain in their initial exposure categories, with censoring due to incident HIV infection or changes to ART regimens, in addition to death or loss to follow-up. Appropriate distributional assumptions will be examined before deploying specific methods, with alternate approaches considered when required. We will assess the potential for missing data to influence findings through sensitivity analyses using multiple imputation with chained equations.[196] For primary outcomes, hypothesis testing will use 2-sided tests with family-wise error rate control (Hommel's method; family-wide significance=0.05);[197-199] secondary and exploratory analyses will use false discovery rate corrections (Benjamini-Hochberg method; $\alpha=0.05$).[200,201]

Analyses will be completed according to aim-specific Statistical Analysis Plans (SAP), approved in advance by study statisticians and including details on statistical and causal

assumptions, variable forms, data reduction methods, and secondary and sensitivity analyses. We will make extensive use of generalized linear mixed models (GLMM) to examine associations between HIV/DTG exposure category and various outcomes of interest, using random effects to account for repeated measures (in our cohort designs including the MC) and/or repeated observations linked to our designs (in case-cohort designs; see below). In the case of multiparity we will use first-born offspring for primary outcomes with secondary analyses including all offspring through within-mother random effects.[202,203]

There is potential for confounding in this observational study making statistical adjustment an important consideration; methods for this are described for each aim, below. Covariates of interest as confounders are aim-specific, in general categories of (i) socio-economic and demographic characteristics, (ii) behaviors related to outcomes of interest (including alcohol, smoking, and/or food consumption in certain analyses), (iii) pre-pregnancy health status including baseline measures of BMI and composition, (iv) maternal HIV disease status and control (including viral load over time), and (v) for infants, *in utero* and delivery measures (including gestational age at birth, SGA, and sex) and maternal measures during pregnancy described above (hypertensive disorders, GDM, multigravidity, etc.). Before the start of analysis, we will specify confounder selection using directed acyclic graphs to distinguish causal intermediates and identify minimum covariate sets for adjustment;[204] secondary analyses will focus on stratified analyses of unconfounded subsets.[205,206]

Here we present primary analyses only for the primary outcome hypotheses. All primary hypotheses will be examined for two definitions of the exposure of interest: (i) the comparison of WLHIV (combining cDTG + iDTG) vs. HIV- women, and (ii) the comparison within WLHIV of iDTG vs. cDTG. Of note, we use T1 (enrollment) measures of key variables to help assess changes over time and to provide insights into measurements among WLHIV not using ART at enrollment, as provided by the iDTG exposure group at T1.

Aim 1. *H1: DTG use in pregnancy will be associated with excess gestational weight gain (GWG) and accrual of adipose tissue mass.* We will estimate GWG (kg/week) and adipose accrual as the difference in adipose (%/week) between T3 and T1; secondary analyses will examine BMI, total body mass, and T2-T1 differences. These continuous constructs will likely be normally distributed, and linear regression models with random intercepts per participant and adjustment for potential confounders will be employed; T1 values will be considered as confounder and/or potential effect modifiers through the use of interaction terms.[207] Secondary analyses will add T2 measures in a repeated measures framework and examine categories GWG per IOM.[208]

Aim 2. *H2: DTG use during pregnancy and postpartum will be associated with adverse maternal metabolic health PP (PP weight retention, adiposity, dysglycemia, IR, and dyslipidemia).* Analysis will begin with investigation of censoring bias using standard methods. PP weight retention will be defined as weight at 12m and 24m PP minus T1 weight (representing pre-pregnancy weight). Adipose accrual/retention will be estimated as described above; insulin resistance and secretion will be based on DIo and Matsuda Index calculations as continuous variables. Lipid values will be analyzed in continuous forms. We will use GLMM with Gaussian and binomial (log-link) families with covariate set adjustment and repeated measures to incorporate outcomes at 12 and 24m PP; analyses will include an interaction term for exposure category by timing of outcome.

Aim 2a. *H2a: Excess GWG and accrual of adipose tissue mass in pregnancy will be associated with adverse metabolic health postpartum.* Building on the analysis from Aim 1, we will examine the associations of GWG and adipose tissue accrual during pregnancy on PP metabolic health using methods described above. The potential role of GWG and adipose tissue accrual as causal mediators will be examined using a series of nested mixed effects models, via the *mediation* package in R.[209-211] This fits one model predicting the

mediator values (here, GWG and adipose accrual) based on HIV/DTG exposure category and potential confounders, and another model for separate PP metabolic health outcomes based on GWG, adipose tissue accrual in pregnancy and HIV/DTG exposure category. Parameters from the two models are used to estimate average causal mediation effects (indirect effects) and average direct effects.[212] Structural nested models via sequential G-estimation to allow for assessment of mediation in the presence of interaction effects and assessment of multiple mediation paths will be considered.[213] Analyses will include potential confounders of mediation effects using directed acyclic graphs (DAGs).[214] Bayesian estimation via Monte Carlo simulation will be used to estimate 95% CI for direct effects and proportion of mediated effect under all models.[215-217]

Aim 3. *H3: In utero DTG-exposure will be associated with adverse neonatal and child metabolic health (increased weight and adipose tissue mass, IR, dyslipidemia).* This analysis will parallel that of Aim 2. Analysis will begin with investigation of censoring bias using standard methods. Neonatal weight and adiposity measured at 2 weeks, including adipose mass and percent body fat from ADP and skinfold anthropometrics, as continuous variables. HOMA-IR and lipid results at 2 years will be considered as continuous variables.

Aim 3a. *H3a: Alterations in neonatal weight and adiposity linked to in utero HIV/DTG exposure will be positively associated with cord blood insulin/C-peptide and adverse child metabolic health.* The analysis here will parallel that of Aim 2a and build on the analysis of Aim 3 above. The association of neonatal weight and adiposity with child metabolic health at 2 years of age will be examined using methods described above, and then their mediating role will be examined using nested mixed effects models to estimate causal direct and indirect effects with bootstrapped 95% CI.

E. POSSIBLE RISKS, PROTECTIONS AGAINST RISKS AND ANTICIPATED BENEFITS

Ethical Review. All ORCHID study protocols, including recruitment, consent, and intervention materials, will be reviewed by the Institutional Review Board (IRB) at Columbia University Irving Medical Center (FWA00002636), Ann and Robert H. Lurie Children's Hospital of Chicago (FWA00001011) and Human Research Ethics Committee (HREC) at the University of Cape Town (FWA00001637). Subsequent to the initial review and approval, the CUMC-IRB and UCT-REC will review progress of the study at least annually.

Potential Risks. The potential risks to participants in the study include:

- *Risk of Discomfort:* Participants will be asked questions about topics that may be sensitive, including demographic, clinical, and behavioral questions, which may cause some discomfort or embarrassment to study participants. Learning one's HIV status may also lead to emotional discomfort, including sadness, anxiety, and emotional distress.
- *Risk associated with phlebotomy and collection of biologic specimens:*
    - Risk associated with phlebotomy: There is a slight risk of physical discomfort to participants associated with collection of blood specimens.
    - Risk associated with fasting blood samples: There is a slight risk that women will experience physical discomfort including hunger, dizziness and nausea secondary to fasting. Some women may feel nauseated after they drink the glucose solution for the OGTT.
    - Risk associated with pre-prandial blood samples: Mothers will be asked to withhold the morning feed for her child on the morning of test. The child may experience hunger and irritability.
    - To minimize risks for bleeding or hematoma during phlebotomy, only trained nurses and phlebotomists with experience in pediatrics will attempt blood draws at all infant/child visits. In addition, blood draws at each study visit will not exceed

the maximum allowed blood volumes in research according to NIH Clinical Center guidance. The NIH recommends a limit of 3 mL/kg per single blood draw and a limit of 7 mL/kg in any 8-week period

- *Risk associated with Air Displacement Plethysmography (ADP):* Women will be asked to sit in a small chamber for three minutes. Some women may experience mild anxiety during the evaluation. Infants will be placed in a small compartment for three minutes and may experience anxiety or fear. For both adult and infant tests, the test chamber has a see-through window that allows the participant to be viewed at all times while inside the test chamber.
- *Risk associated with Indirect Calorimetry:* Women will be asked to fast overnight (see above risk associated with fasting); Participants read or watch television; after a 20min period of initial quiet rest, participants will choose to wear either a low-profile facemask or canopy hood for another 20min while reclining/resting. They may experience discomfort wearing the mask or hood or while reclining.
- *Risk of contracting COVID-*19: there may be risk of contracting COVID-19 while participant in study activities. All study processes will be conducted with strict adherence to COVID-19 protection protocols.
- *Risk of Loss of Confidentiality:* There is a risk of loss of confidentiality of information provided during the study, including participant HIV status through conduct of interviews and by nature of participation in the study.
- *Risk of Social Harms:* Participants may experience social stigma or incur harm because of identification in the community with a HIV-related study and its research staff. Social harms due to study participation may include loss of privacy, social stigma from perception of having HIV infection or being at risk for HIV infection.

All participants will be informed of these risks as part of informed consent. We planned specific steps in study design and conduct (below) to minimize the possibility of those risks. Those steps draw directly from our prior experiences conducting clinical studies involving pregnant women and their children in South Africa, Botswana, Cameroon, and the United States.

Protections Against Risk. The protections against risk to participants in the study include:

- *Risk of Discomfort:* We will minimize the potential psychological distress to participants in the study through several means:
    - Informed consent documents will mention the sensitive nature of some study assessments, including questions about health, food intake, substance use, finances, social support, and HIV status.
    - Reminding participants that they may decline to respond to any item(s) during study interviews and may terminate interviews and study participation at any time without affecting the quality of health care received.
    - Training the research team to recognize the common symptoms and signs of psychological distress, including anxiety, depression, and possible suicidality, in participants.
    - Ensuring that there are appropriate management mechanisms for participants found to experience any form of distress, including:
        - For very mild forms of distress, immediate management through counselling by research staff.
        - For moderate and severe forms of distress, immediate referral to the mental health care service at the Gugulethu or Mitchell's Plain primary health care center (staffed by a full-time psychiatric nursing sister and overseen by part-time psychologist and trainee psychiatrist).

- Referral to support services relevant to specific concerns, such as NGOs providing services for women who experience domestic violence or substance use counselling centers. The Desmond Tutu HIV Center maintains active relationships with the major support services operating in the Gugulethu and Mitchell's Plain community and our experience referring patients to these support services has been positive.
- We will maintain an up to date list of referral organizations for mental health support, substance use, alcoholism, and interpersonal violence for participants to receive additional counselling, social, and medical services.

Any instance of psychosocial distress requiring referral will be recorded as an adverse event during the study and will be reviewed by the study team. The Site Coordinator and Dr Myer will be responsible for monitoring the referral process and outcomes for all participants identified as having experienced psychosocial distress.

- *Risk associated with collection of biologic specimens:*
  - Risk associated with phlebotomy. We will minimize risks associated with phlebotomy for venous blood collection through:
    - Standard training of research nurse in blood collection, using an established SOP for phlebotomy;
    - Ensuring availability of appropriate phlebotomy equipment (including alcohol swabs and supplies for the Vacutainer system) throughout the study; and
    - Providing the research nurse working in the study measurement team with access to a doctor to assist with difficult cases.

Any significant complications of phlebotomy will be referred to a senior study physician for assessment and management, including referral to specialist care, as required.

  - Risk associated with fasting or pre-prandial blood samples. We will minimize the discomfort from fasting or pre-prandial samples and monitor for signs of light-headedness or dizziness upon arrival after an overnight fast and throughout the entirety of the OGTT (for women).

- *Risk associated with Air Displacement Plethysmography:* We will minimize the mild anxiety or fear associated with ADP testing through careful preparation of participants, with pictorial and written information (including wall posters and pamphlets) explaining the ADP measurement process for adults and children. In addition, we will provide an opportunity for participants to witness an ADP measurement taking place (either in real time or a video) if they would like, as well as to ask any questions about the process. In our experience, only a small minority of individuals may encounter concerns around ADP measurement, and these are easily addressed through these steps. In addition, it is important to note that ADP avoids the safety concerns around alternative measures such as DXA in pregnant women and infants.

- *Risk associated with Indirect Calorimetry.* Similarly to the steps related to ADP measurement, we will minimize the risks associated with indirect calorimetry through careful preparation of participants, with pictorial and written information (including wall posters and pamphlets) explaining the indirect calorimetry measurement process. In addition, we will provide an opportunity for participants to witness indirect calorimetry measurement taking place (either in real time or a video) if they would like, as well as to ask any questions about the process. In our experience, only a small minority of individuals may encounter concerns around this measurement, and these are easily addressed through these steps.

- *Risk of contracting COVID-*19: This risk will be minimised because everyone attending Gugulethu CHC including patients and staff members are screened for COVID-19 symptoms prior to entering the clinic and are not allowed in if they have been exposed to a patient with Coronavirus or have a fever.
    - Staff safety: all staff will always have their masks on and they'll frequently sanitise with alcohol-based hand disinfectant. Staff will also be trained on how to disinfect surfaces (with bleach solution) including chairs, tables, phones, keyboards, and tablets before and after seeing each participant. There is a dedicated cleaner who will also ensure that surfaces are cleaned frequently.
    - Participant safety: When participants arrive in our research place, we will provide them with a disposable surgical mask if they are not wearing any mask and a hand sanitizer to disinfect their hands. Throughout the visit, participants will use readily available hand sanitizer before and after entering the study room (and before/after eating/using toilets). Social distancing will be adhered to between staff and participants. For follow-up visits, participants will be contacted by study staff a day before their visit to check whether they have any symptoms of Covid-19, if so they will be advised not to come for a study visit but to visit their nearest clinic for testing.

- *Risk of Loss of Confidentiality:* We will minimize the risk of any loss of confidentiality throughout study design and conduct. These features will be included in the study manual of procedures and specific SOPs.
    - All personnel involved in data collection and management will undergo specific training for the study in confidentiality and related patient protection issues (in addition to routine GCP and Human Subjects Protection training).
    - Following standard practice, all patient and study related information will be kept in locked cabinets at either the study office in Gugulethu, Mitchell's Plain, or UCT.
    - Anonymous participant identification numbers will be used on all study documents. Collection of participant names and other identifiers will be restricted to informed consent documents, patient tracing materials, and a study identification key (all of which will be kept in a locked cabinet at UCT separate from other study documentation and accessible only by the project manager and the PIs.). No study forms will include participant names, including forms that may reflect HIV status of women, partners, or their children (including HIV test results).
    - All electronic records will be kept in password protected files. All electronic communications of study data will be conducted through password-protected, encrypted files. All data will be stored at the University of Cape Town will be within a firewall protected SQL server.

  We have refined protocols to ensure safeguarding of participant identity during previous studies and have not yet encountered a breach in confidentiality due to study procedures including screening and recruitment, data collection, data management, or other study procedures. Note that informed consent documents will describe confidentiality and the legal limits of confidentiality, clarifying that any direct threat to safety identified (e.g., suicidality, homicidally, or abuse) will require notification to the relevant authorities per South African law.

- *Risk of Social Harms:* To minimize the risks of social harms, we will train the study team to detect potential social harms, including basic training on the common symptoms and signs of psychological distress, including anxiety, depression, and

possible suicidality, in participants. We will ensure that there are appropriate management mechanisms for participants found to experience any form of social harm or distress, during study participation, including:

- For very mild forms of distress, immediate management through counselling by study personnel.
- For moderate and severe forms of distress, immediate referral to mental health care services at primary health care centers in the community.
- For other concerns, including domestic violence or substance abuse, referral to support services such as NGOs that provide services in this community.

As part of previous NICHD-funded research our group has developed an SOP detailing referrals for psychological and other concerns, and all study staff will receive training on this SOP prior to the start of the study. Our group maintains active relationships with the major support services operating in this community, and our previous experience referring patients to these support services has been positive. The site coordinator and project manager will be responsible for monitoring the referral process and outcomes for all participants identified as having experienced psychosocial distress.

Additional Protection for Pregnant Women. Our study seeks to better understand the impact of DTG in pregnancy and its obesogenic effects on the metabolic health of women living with HIV (WLHIV) and their children, compared to women without HIV and their children. Therefore, to achieve our study aims, we are including pregnant women. Under 45 CFR 46 Subpart B, this study is allowed to involve pregnant women because study procedures do not involve the testing of any new drugs or procedures and only include use of methods that are considered to be safe. Therefore, we do not anticipate any additional risk to pregnant women or to their fetus from participating in the study. In addition, all women will be under the supervision of a trained physician and because this study takes place inside a Western Cape Provincial Government clinic medical personnel will be readily available should urgent medical events arise unrelated to study procedures (i.e. labor pains; See Medical or Professional Intervention in Event of Adverse Event section below). As a research project, we will have no part in making medical decisions regarding the health of the pregnant woman or her fetus, including but not limited to determination of the viability of the fetus or need for inducing labor. Additionally, we will have no part in making any diagnoses related to pregnancy or other conditions such as preeclampsia or cytomegalovirus. Although we will be conducting a study about DTG-associated metabolic health outcomes during pregnancy and postpartum, we will not change medication prescriptions or impact clinical care. If a participant complains about their current prescriptions or states that they would like to stop their prescription, they will be told by the study staff to continue taking their medications as prescribed and instructed to bring these concerns to their antenatal care physician for the protection of themselves and their fetus. Because this study will be conducted in a health care setting, study staff will remind study participants that we are not related to their pregnancy or HIV care in any capacity, when necessary.

Medical or Professional Intervention in Event of Adverse Event**.** Although we do not anticipate any events in need of medical intervention associated with our study activities, due to our special population of pregnant and postpartum women, some of whom are living with HIV, we do recognize the need for safeguards. All participants will be under the care of a physician as our study site is based at a Western Cape Provincial Government clinic. We will emphasize that research staff members are not medical providers and medical concerns should be discussed with the participant's doctor. All women screening positive for a hypertensive disorder or gestational diabetes during the study will be given information about these complications during pregnancy, the importance of appropriate follow-up care and clinical management, and will be referred for specialized maternity care, which is available in the Western Cape at no cost. If a medical event does arise during our research activities (e.g. labor pains), with the participant's permission, we will consult with the medical staff in

the clinic and transfer her to their care, if necessary. All adverse events will be immediately reported to the PI. Serious adverse events will be reported to the University of Cape Town HREC and Columbia and Northwestern University's IRBs by phone and followed by a written report of notification within 24 hours of the event. The PI will also notify the NIH Program officer and will report adverse event in writing to the NIH funding institute. The Adverse event will also be reviewed by Prof Myer to determine subsequent actions and if revisions to current protocols are needed.

Benefits. There are limited direct benefits of participation. Participants will have a frequent clinical and laboratory assessments that they may not otherwise receive including first and third trimester USG and OGTT, fasting lipids and for women living with HIV frequent HIV monitoring (CD4, HIV viral load testing, etc.). These assessments may be useful in identifying health conditions including hypertensive disorders, gestational diabetes warranting further evaluation or intervention. Women without HIV will also have regular testing for HIV and new HIV infections will be detected early: women who test HIV positive will be referred for immediate ART initiation. Results of all laboratory studies will be made available to primary health providers. In addition, women participating in the study may benefit from improved literacy about health, diet, and weight gain.

There is indirect benefit to society in helping to better understand the impact of DTG on the metabolic health of women living with HIV and their children.

Data Safety and Monitoring. All study protocols (including recruitment strategies, consent procedures, interviews and assessments, and ongoing adverse event monitoring) will be reviewed and subject to approval by the Columbia and Ann & Robert H. Lurie Hospital of Chicago/Northwestern University's IRBs and the University of Cape Town HREC. This protocol complies with the Declaration of Helsinki (2013) and the South African Department of Health: Ethics in Health Research: Principles, Structures, and Processes (2004). To maintain compliance with the NIH Data and Safety Monitoring Policy, we propose the following monitoring plan:

The PI (L Myer) will conduct internal monitoring of the safety of human subjects, with consultation with E Abrams and J Jao. Non-emergency issues will be discussed at our weekly research team meetings (Study coordinator and research assistants) that are held to address study progress, recruitment, data collection, retention, and other factors related to human subjects. Local PI Prof Myer will provide oversight in these research activities. We will also create emergency protocols for serious adverse events and serious ethical issues. These protocols will outline steps to take if a potential adverse event occurs and research staff members will be trained in these protocols (see section on 'Medical or Professional Intervention in Event of Adverse Event' above).

F. EMERGENCY CARE AND INSURANCE

This study falls under the University of Cape Town's no fault insurance policy to cover injuries incurred in research not sponsored by a pharmaceutical company.

G. DISSEMINATION

Throughout this study, we will disseminate findings to local health departments and non-governmental organizations through study briefs and PI's (Abrams, Myer, Jao) will present at domestic/international conferences. We will also disseminate data through publishing. Authors on any publications will consist of the entire investigative team.

F. REFERENCES

1. Puoane T, Steyn K, Bradshaw D, et al. Obesity in South Africa: the South African demographic and health survey. Obes Res 2002;10:1038-48
2. Group NAS, Kouanfack C, Mpoudi-Etame M, et al. Dolutegravir-Based or Low-Dose Efavirenz-Based Regimen for the Treatment of HIV-1. N Engl J Med 2019;381:816-26
3. Venter WDF, Moorhouse M, Sokhela S, et al. Dolutegravir plus Two Different Prodrugs of Tenofovir to Treat HIV. N Engl J Med 2019;381:803-15
4. Longmore DK, Barr ELM, Lee IL, et al. Maternal body mass index, excess gestational weight gain, and diabetes are positively associated with neonatal adiposity in the Pregnancy and Neonatal Diabetes Outcomes in Remote Australia (PANDORA) study. Pediatr Obes 2019;14:e12490
5. Catalano PM, Farrell K, Thomas A, et al. Perinatal risk factors for childhood obesity and metabolic dysregulation. Am J Clin Nutr 2009;90:1303-13.PMC2762159
6. Goldstein RF, Abell SK, Ranasinha S, et al. Association of Gestational Weight Gain With Maternal and Infant Outcomes: A Systematic Review and Meta-analysis. JAMA 2017;317:2207-25.PMC5815056
7. Macdonald-Wallis C, Tilling K, Fraser A, Nelson SM, Lawlor DA. Gestational weight gain as a risk factor for hypertensive disorders of pregnancy. Am J Obstet Gynecol 2013;209:327 e1-17.PMC3807791
8. Widen EM, Whyatt RM, Hoepner LA, et al. Excessive gestational weight gain is associated with long-term body fat and weight retention at 7 y postpartum in African American and Dominican mothers with underweight, normal, and overweight prepregnancy BMI. Am J Clin Nutr 2015;102:1460-7.PMC4658466
9. Catalano PM, Shankar K. Obesity and pregnancy: mechanisms of short term and long term adverse consequences for mother and child. BMJ 2017;356:j1.PMC6888512
10. WHO Fact Sheet on Overweight and Obesity 2018. Accessed at https://www.who.int/news-room/fact-sheets/detail/obesity-and-overweight on December 18, 2019.
11. WHO Global Health Observatory Data. Prevalence of Overweight among Adults 18 years and Older in 2016. Accessed at https://www.who.int/gho/ncd/risk_factors/overweight/en/ on December 18, 2019. .
12. Lavie CJ, Milani RV, Ventura HO. Obesity and cardiovascular disease: risk factor, paradox, and impact of weight loss. J Am Coll Cardiol 2009;53:1925-32
13. Abdelaal M, le Roux CW, Docherty NG. Morbidity and mortality associated with obesity. Ann Transl Med 2017;5:161.PMC5401682
14. Flegal KM, Kit BK, Orpana H, Graubard BI. Association of all-cause mortality with overweight and obesity using standard body mass index categories: a systematic review and meta-analysis. JAMA 2013;309:71-82.PMC4855514
15. UNAIDS South Africa. Accessed at https://www.unaids.org/en/regionscountries/countries/southafrica on December 20, 2019.
16. Ben-Shlomo Y, Kuh D. A life course approach to chronic disease epidemiology: conceptual models, empirical challenges and interdisciplinary perspectives. Int J Epidemiol 2002;31:285-93
17. WHO Update of recommendations on first- and second-line antiretroviral regimens 2019. Accessed at https://www.who.int/hiv/pub/arv/arv-update-2019-policy/en/ on December 20, 2019. .
18. Dugdale CM, Ciaranello AL, Bekker LG, et al. Risks and Benefits of Dolutegravir- and Efavirenz-Based Strategies for South African Women With HIV of Child-Bearing Potential: A Modeling Study. Ann Intern Med 2019;170:614-25.PMC6736740
19. Phillips AN, Venter F, Havlir D, et al. Risks and benefits of dolutegravir-based antiretroviral drug regimens in sub-Saharan Africa: a modelling study. Lancet HIV 2019;6:e116-e27.PMC6361866
20. UNAIDS. The Gap Report: Children and Pregnant Women Living with HIV. Accessed at http://wwwunaidsorg/sites/default/files/media_asset/09_ChildrenandpregnantwomenlivingwithHIVpdf on September 29, 2015. Geneva2014.
21. Bhagwat P, Ofotokun I, McComsey GA, et al. Changes in Waist Circumference in HIV-Infected Individuals Initiating a Raltegravir or Protease Inhibitor Regimen: Effects of Sex and Race. Open forum infectious diseases 2018;5:ofy201.PMC6239079
22. Bourgi K, Rebeiro PF, Turner M, et al. Greater Weight Gain in Treatment-naive Persons Starting Dolutegravir-based Antiretroviral Therapy. Clin Infect Dis 2020;70:1267-74
23. Norwood J, Turner M, Bofill C, et al. Brief Report: Weight Gain in Persons With HIV Switched From Efavirenz-Based to Integrase Strand Transfer Inhibitor-Based Regimens. J Acquir Immune Defic Syndr 2017;76:527-31.PMC5680113
24. Lake JE, Wu K, Bares SH, et al. Risk Factors for Weight Gain Following Switch to Integrase Inhibitor-Based Antiretroviral Therapy. Clin Infect Dis 2020
25. Eckard AR, McComsey GA. Weight gain and integrase inhibitors. Curr Opin Infect Dis 2020;33:10-9
26. Kerchberger AM, Sheth AN, Angert CD, et al. Weight Gain Associated with Integrase Stand Transfer Inhibitor Use in Women. Clin Infect Dis 2019
27. Lamorde M, Atwiine M, Owarwo NC, et al. Dolutegravir-associated hyperglycaemia in patients with HIV. Lancet HIV 2020
28. Bourgi K, Jenkins CA, Rebeiro PF, et al. Weight gain among treatment-naive persons with HIV starting integrase inhibitors compared to non-nucleoside reverse transcriptase inhibitors or protease inhibitors in a large observational cohort in the United States and Canada. J Int AIDS Soc 2020;23:e25484.PMC7159248
29. Waitt C, Orrell C, Walimbwa S, et al. Safety and pharmacokinetics of dolutegravir in pregnant mothers with HIV infection and their neonates: A randomised trial (DolPHIN-1 study). PLoS medicine 2019;16:e1002895.PMC6754125 following competing interests: ML declared research grants from ViiV, Janssen and personal fees from Mylan.
30. Mulligan N, Best BM, Wang J, et al. Dolutegravir pharmacokinetics in pregnant and postpartum women living with HIV. AIDS 2018;32:729-37.PMC5854536
31. Truong YN, Yee LM, Caughey AB, Cheng YW. Weight gain in pregnancy: does the Institute of Medicine have it right? Am J Obstet Gynecol 2015;212:362 e1-8
32. Johnson J, Clifton RG, Roberts JM, et al. Pregnancy outcomes with weight gain above or below the 2009 Institute of Medicine guidelines. Obstet Gynecol 2013;121:969-75.PMC3971915
33. Egan AM, Dennedy MC, Al-Ramli W, Heerey A, Avalos G, Dunne F. ATLANTIC-DIP: excessive gestational weight gain and pregnancy outcomes in women with gestational or pregestational diabetes mellitus. J Clin Endocrinol Metab 2014;99:212-9
34. McClure CK, Catov JM, Ness R, Bodnar LM. Associations between gestational weight gain and BMI, abdominal adiposity, and traditional measures of cardiometabolic risk in mothers 8 y postpartum. Am J Clin Nutr 2013;98:1218-25.PMC3798077

35. Berggren EK, Groh-Wargo S, Presley L, Hauguel-de Mouzon S, Catalano PM. Maternal fat, but not lean, mass is increased among overweight/obese women with excess gestational weight gain. Am J Obstet Gynecol 2016;214:745 e1-5.PMC4884531
36. Vesco KK, Dietz PM, Rizzo J, et al. Excessive gestational weight gain and postpartum weight retention among obese women. Obstet Gynecol 2009;114:1069-75
37. Al Mamun A, Mannan M, O'Callaghan MJ, Williams GM, Najman JM, Callaway LK. Association between gestational weight gain and postpartum diabetes: evidence from a community based large cohort study. PLoS One 2013;8:e75679.PMC3862846
38. Reynolds RM, Osmond C, Phillips DI, Godfrey KM. Maternal BMI, parity, and pregnancy weight gain: influences on offspring adiposity in young adulthood. J Clin Endocrinol Metab 2010;95:5365-9
39. Catalano PM, Presley L, Minium J, Hauguel-de Mouzon S. Fetuses of obese mothers develop insulin resistance in utero. Diabetes Care 2009;32:1076-80.PMC2681036
40. Josefson JL, Catalano PM, Lowe WL, et al. The Joint Associations of Maternal BMI and Glycemia with Childhood Adiposity. J Clin Endocrinol Metab 2020
41. Castillo-Laura H, Santos IS, Quadros LC, Matijasevich A. Maternal obesity and offspring body composition by indirect methods: a systematic review and meta-analysis. Cad Saude Publica 2015;31:2073-92
42. Caniglia E SR, Diseko M, Wylie B, Zera C, Davey S, Isaacson A, Mayondi G, Mabuta J, Luckett R, Makhema J, Mmalane M, Lockman S, Zash R, TheTsepamo Study. IAS July 2019, Mexico City Abs. LBPEB14. Weight gain during pregnancy among women initiating dolutegravir in Botswana, Abstract LBPEB14. Presented at the International AIDS Society (IAS), July 2019, Mexico City, Mexico. 2019
43. Chinula L, Brummel S, Ziemba L, Stranix-Chibanda L, Coletti A, Krotje C, Jean-Philippe P, Fairlie L, Vhembo T, IMPAACT. SAFETY AND EFFICACY OF DTG VS EFV AND TDF VS TAF IN PREGNANCY: IMPAACT 2010 TRIAL. Abstract 130. Presented at the Conference on Retroviruses and Opportunistic Infections (CROI), March 2020, Boston, MA, USA. 2020
44. Bengston AM, Le Roux SM, Phillips TK, Brittain K, Zerbe A, Malaba T, Petro G, Madlala HP, Abrams EJ, Myer. Does HIV-infection modify the relationship between pre-pregnancy body mass index and adverse birth outcomes: evidence from a South African cohort with a high burden of HIV and obesity. Pediatric and Perinatal Epidemiology, in press 2019
45. Madlala HP, Malaba TF, Newell ML, Myer L. Obesity, gestational weight gain and adverse birth outcomes in South African women. Poster 3295. Accepted at the Conference on Retroviruses and Opportunistic Infections (CROI), Mar 2020, Boston, MA, USA.
46. Jao J, Sun S, Legbedze J, Jacobson D, Mmasa KN, Kgole S, Masasa G, Makhema J, Moyo S, Mmalane M, Banda F, Nkomo B, Gerschenson M, Abrams EJ, Powis KM. . Dolutegravir Use is Associated with Higher Postpartum Weight Compared to Efavirenz. Poster 2777. Accepted at the Conference on Retroviruses and Opportunistic Infections (CROI), March 2020, Boston, MA, USA.
47. Malaba T, Chen T, Kintu K, Papamichael C, Reynolds H, Nakibuka N, Waitt C, Hodel EM, Colbers A, Orrell C, Wang D, Lamorde M, Khoo S, Myer L. Postpartum weight changes in women initiating DTG vs EFV in pregnancy: DolPHIN-2. Poster 3202. Accepted for presentation at the Conference on Retroviruses and Opportunistic Infections (CROI), March 2020, Boston, MA, USA.
48. Meng Y, Groth SW, Stewart P, Smith JA. An Exploration of the Determinants of Gestational Weight Gain in African American Women: Genetic Factors and Energy Expenditure. Biol Res Nurs 2018;20:118-25.PMC5896561
49. Berggren EK, O'Tierney-Ginn P, Lewis S, Presley L, De-Mouzon SH, Catalano PM. Variations in resting energy expenditure: impact on gestational weight gain. Am J Obstet Gynecol 2017;217:445 e1- e6.PMC5614817
50. Wrottesley SV, Pisa PT, Norris SA. The Influence of Maternal Dietary Patterns on Body Mass Index and Gestational Weight Gain in Urban Black South African Women. Nutrients 2017;9.PMC5537846
51. Agostini CO, Zoche E, Correa RDS, Chaves EBM, Corleta HVE, Bosa VL. Contribution of Ultra-processed Food to the Daily Food Intake of HIV-positive and HIV-Negative Women during Pregnancy. Rev Bras Ginecol Obstet 2019;41:588-96
52. Mittelsteadt AL, Hileman CO, Harris SR, Payne KM, Gripshover BM, McComsey GA. Effects of HIV and antiretroviral therapy on resting energy expenditure in adult HIV-infected women-a matched, prospective, cross-sectional study. J Acad Nutr Diet 2013;113:1037-43
53. Kosmiski LA, Kuritzkes DR, Sharp TA, et al. Total energy expenditure and carbohydrate oxidation are increased in the human immunodeficiency virus lipodystrophy syndrome. Metabolism 2003;52:620-5
54. Kosmiski L. Energy expenditure in HIV infection. Am J Clin Nutr 2011;94:1677S-82S.PMC3226020
55. Pernerstorfer-Schoen H, Schindler K, Parschalk B, et al. Beneficial effects of protease inhibitors on body composition and energy expenditure: a comparison between HIV-infected and AIDS patients. AIDS 1999;13:2389-96
56. Eckard A, Sattar A, Yu J, Hughes HY, Labbato D, Rodgers TO , Kosco JC, and McComsey GA. FAT GAINS OCCUR AFTER ART WITHOUT CHANGES IN METABOLIC RATE OR CALORIC INTAKE. Abstract 667. Presented at CROI, March 8-11, 2020, Boston, MA, USA.
57. Logan CA, Bornemann R, Koenig W, et al. Gestational Weight Gain and Fetal-Maternal Adiponectin, Leptin, and CRP: results of two birth cohorts studies. Sci Rep 2017;7:41847.PMC5288774
58. Baratto I, Daher S, Lobo TF, Araujo Junior E, Guazzelli CAF. Adiponectin and leptin serum levels in normal adolescent pregnancies. J Matern Fetal Neonatal Med 2019:1-6
59. Highman TJ, Friedman JE, Huston LP, Wong WW, Catalano PM. Longitudinal changes in maternal serum leptin concentrations, body composition, and resting metabolic rate in pregnancy. Am J Obstet Gynecol 1998;178:1010-5
60. Weisberg SP, McCann D, Desai M, Rosenbaum M, Leibel RL, Ferrante AW, Jr. Obesity is associated with macrophage accumulation in adipose tissue. J Clin Invest 2003;112:1796-808.PMC296995
61. Kern PA, Ranganathan S, Li C, Wood L, Ranganathan G. Adipose tissue tumor necrosis factor and interleukin-6 expression in human obesity and insulin resistance. American journal of physiology Endocrinology and metabolism 2001;280:E745-51
62. Fjeldborg K, Christiansen T, Bennetzen M, H JM, Pedersen SB, Richelsen B. The macrophage-specific serum marker, soluble CD163, is increased in obesity and reduced after dietary-induced weight loss. Obesity (Silver Spring) 2013;21:2437-43
63. Cinkajzlova A, Lacinova Z, Klouckova J, et al. An alternatively activated macrophage marker CD163 in severely obese patients: the influence of very low-calorie diet and bariatric surgery. Physiol Res 2017;66:641-52
64. Conley LJ, Bush TJ, Rupert AW, et al. Obesity is associated with greater inflammation and monocyte activation among HIV-infected adults receiving antiretroviral therapy. AIDS 2015;29:2201-7
65. Lake JE, McComsey GA, Hulgan T, et al. Switch to raltegravir decreases soluble CD14 in virologically suppressed overweight women: the Women, Integrase and Fat Accumulation Trial. HIV Med 2014;15:431-41.PMC4107004
66. El Kamari V, Moser C, Hileman CO, et al. Lower Pretreatment Gut Integrity Is Independently Associated With Fat Gain on Antiretroviral Therapy. Clin Infect Dis 2019;68:1394-401.PMC6599164

67. McMahon C, Trevaskis JL, Carter C, et al. Lack of an association between clinical INSTI-related body weight gain and direct interference with MC4 receptor (MC4R), a key central regulator of body weight. PLoS One 2020;15:e0229617.PMC7048285
68. Domingo P, Villarroya F, Giralt M, Domingo JC. Potential role of the melanocortin signaling system interference in the excess weight gain associated to some antiretroviral drugs in people living with HIV. Int J Obes (Lond) 2020
69. Cone RD. Anatomy and regulation of the central melanocortin system. Nat Neurosci 2005;8:571-8
70. Saeed S, Bonnefond A, Manzoor J, et al. Genetic variants in LEP, LEPR, and MC4R explain 30% of severe obesity in children from a consanguineous population. Obesity (Silver Spring) 2015;23:1687-95
71. Gorwood J, Bourgeois C, Pourcher V, et al. The integrase inhibitors dolutegravir and raltegravir exert pro-adipogenic and profibrotic effects and induce insulin resistance in human/simian adipose tissue and human adipocytes. Clin Infect Dis 2020
72. Goedecke JH, Levitt NS, Evans J, et al. The role of adipose tissue in insulin resistance in women of African ancestry. J Obes 2013;2013:952916.PMC3557633
73. Vernochet C, Damilano F, Mourier A, et al. Adipose tissue mitochondrial dysfunction triggers a lipodystrophic syndrome with insulin resistance, hepatosteatosis, and cardiovascular complications. FASEB J 2014;28:4408-19.PMC4202105
74. Woo CY, Jang JE, Lee SE, Koh EH, Lee KU. Mitochondrial Dysfunction in Adipocytes as a Primary Cause of Adipose Tissue Inflammation. Diabetes Metab J 2019;43:247-56.PMC6581541
75. Bluher M. Adipose tissue inflammation: a cause or consequence of obesity-related insulin resistance? Clin Sci (Lond) 2016;130:1603-14
76. Reilly SM, Saltiel AR. Adapting to obesity with adipose tissue inflammation. Nat Rev Endocrinol 2017;13:633-43
77. Kotze-Horstmann LM, Keswell D, Adams K, Dlamini T, Goedecke JH. Hypoxia and extra-cellular matrix gene expression in adipose tissue associates with reduced insulin sensitivity in black South African women. Endocrine 2017;55:144-52
78. Goedecke JH, Mtintsilana A, Dlamini SN, Kengne AP. Type 2 diabetes mellitus in African women. Diabetes Res Clin Pract 2017;123:87-96
79. Evans J, Goedecke JH, Soderstrom I, et al. Depot- and ethnic-specific differences in the relationship between adipose tissue inflammation and insulin sensitivity. Clin Endocrinol (Oxf) 2011;74:51-9
80. Goedecke JH, Keswell D, Weinreich C, et al. Ethnic differences in hepatic and systemic insulin sensitivity and their associated determinants in obese black and white South African women. Diabetologia 2015;58:2647-52.PMC4668114
81. Goedecke JH, Dave JA, Faulenbach MV, et al. Insulin response in relation to insulin sensitivity: an appropriate beta-cell response in black South African women. Diabetes Care 2009;32:860-5.PMC2671086
82. Goedecke JH, Levitt NS, Lambert EV, et al. Differential effects of abdominal adipose tissue distribution on insulin sensitivity in black and white South African women. Obesity (Silver Spring) 2009;17:1506-12
83. Goedecke JH, Olsson T. Pathogenesis of type 2 diabetes risk in black Africans: a South African perspective title. J Intern Med 2020
84. Hulgan T, Ramsey BS, Koethe JR, et al. Relationships Between Adipose Mitochondrial Function, Serum Adiponectin, and Insulin Resistance in Persons With HIV After 96 Weeks of Antiretroviral Therapy. J Acquir Immune Defic Syndr 2019;80:358-66.PMC6375746
85. Agarwal P, Morriseau TS, Kereliuk SM, Doucette CA, Wicklow BA, Dolinsky VW. Maternal obesity, diabetes during pregnancy and epigenetic mechanisms that influence the developmental origins of cardiometabolic disease in the offspring. Crit Rev Clin Lab Sci 2018;55:71-101
86. Shi X, Huang P, Wang L, et al. Maternal postload 1-hour glucose level during pregnancy and offspring's overweight/obesity status in preschool age. BMJ Open Diabetes Res Care 2020;8.PMC7039585
87. Perng W, Hockett CW, Sauder KA, Dabelea D. In utero exposure to gestational diabetes mellitus and cardiovascular risk factors in youth: A longitudinal analysis in the EPOCH cohort. Pediatr Obes 2020;15:e12611
88. Leddy MA, Power ML, Schulkin J. The impact of maternal obesity on maternal and fetal health. Rev Obstet Gynecol 2008;1:170-8.PMC2621047
89. Tenenbaum-Gavish K, Hod M. Impact of maternal obesity on fetal health. Fetal Diagn Ther 2013;34:1-7
90. Badon SE, Dyer AR, Josefson JL, Group HSCR. Gestational weight gain and neonatal adiposity in the Hyperglycemia and Adverse Pregnancy Outcome study-North American region. Obesity (Silver Spring) 2014;22:1731-8.PMC4100536
91. Lowe WL, Jr., Scholtens DM, Lowe LP, et al. Association of Gestational Diabetes With Maternal Disorders of Glucose Metabolism and Childhood Adiposity. JAMA 2018;320:1005-16.PMC6143108
92. HAPO Study Cooperative Research Group. Hyperglycemia and Adverse Pregnancy Outcome (HAPO) Study: associations with neonatal anthropometrics. Diabetes 2009;58:453-9.PMC2628620
93. Lowe WL, Jr., Scholtens DM, Kuang A, et al. Hyperglycemia and Adverse Pregnancy Outcome Follow-up Study (HAPO FUS): Maternal Gestational Diabetes Mellitus and Childhood Glucose Metabolism. Diabetes Care 2019;42:372-80.PMC6385693
94. Sonagra AD, Biradar SM, K D, Murthy DSJ. Normal pregnancy- a state of insulin resistance. Journal of clinical and diagnostic research : JCDR 2014;8:CC01-3.PMC4290225
95. Del Prato S, Tiengo A. The importance of first-phase insulin secretion: implications for the therapy of type 2 diabetes mellitus. Diabetes Metab Res Rev 2001;17:164-74
96. Choi SM, Tucker DF, Gross DN, et al. Insulin regulates adipocyte lipolysis via an Akt-independent signaling pathway. Mol Cell Biol 2010;30:5009-20.PMC2953052
97. Gower BA, Hunter GR, Chandler-Laney PC, Alvarez JA, Bush NC. Glucose metabolism and diet predict changes in adiposity and fat distribution in weight-reduced women. Obesity (Silver Spring) 2010;18:1532-7.PMC3070365
98. Gower BA, Alvarez JA, Bush NC, Hunter GR. Insulin sensitivity affects propensity to obesity in an ethnic-specific manner: results from two controlled weight loss intervention studies. Nutr Metab (Lond) 2013;10:3.PMC3571978
99. Sanchez-Campillo M, Ruiz-Palacios M, Ruiz-Alcaraz AJ, et al. Child Head Circumference and Placental MFSD2a Expression Are Associated to the Level of MFSD2a in Maternal Blood During Pregnancy. Front Endocrinol (Lausanne) 2020;11:38.PMC7012934
100. Dentin R, Benhamed F, Pegorier JP, et al. Polyunsaturated fatty acids suppress glycolytic and lipogenic genes through the inhibition of ChREBP nuclear protein translocation. J Clin Invest 2005;115:2843-54.PMC1224299
101. Jump DB. N-3 polyunsaturated fatty acid regulation of hepatic gene transcription. Current opinion in lipidology 2008;19:242-7.PMC2764370
102. Jao J, Sun S, Kraus T, Kirmse B, Geffner M, Qiu Y, Arpadi S, Abrams EJ, LeRoith D, Sperling RS, Kurland IJ. . Distinct cord C-peptide, adipokine, and lipidomic signatures by in utero HIV exposure. Presented at the Conference on Retroviruses and Opportunistic Infections (CROI), March 2020, Boston, MA, USA.

103. Ortega-Senovilla H, Schaefer-Graf U, Herrera E. Pregnant women with gestational diabetes and with well controlled glucose levels have decreased concentrations of individual fatty acids in maternal and cord serum. Diabetologia 2020;63:864-74
104. Balogun K, Balmert L, Jao J, Sun S, Bazinet R, and Serghides L. Maternal and cord plasma bioactive eicosanoid profiles differ in HIV+ and HIV- women. Presented at the Conference on Retroviruses and Opportunistic Infections (CROI), March 2020, Boston, MA, USA.
105. Jao J, Kirmse B, Yu C, et al. Lower Preprandial Insulin and Altered Fuel Use in HIV/Antiretroviral-Exposed Infants in Cameroon. J Clin Endocrinol Metab 2015;100:3260-9.PMC4570172
106. Makela J, Linderborg K, Niinikoski H, Yang B, Lagstrom H. Breast milk fatty acid composition differs between overweight and normal weight women: the STEPS Study. Eur J Nutr 2013;52:727-35
107. Armand M, Bernard JY, Forhan A, Heude B, Charles MA, group Em-ccs. Maternal nutritional determinants of colostrum fatty acids in the EDEN mother-child cohort. Clin Nutr 2018;37:2127-36
108. Rudolph MC, Young BE, Lemas DJ, et al. Early infant adipose deposition is positively associated with the n-6 to n-3 fatty acid ratio in human milk independent of maternal BMI. Int J Obes (Lond) 2017;41:510-7.PMC5380514
109. Young BE, Levek C, Reynolds RM, et al. Bioactive components in human milk are differentially associated with rates of lean and fat mass deposition in infants of mothers with normal vs. elevated BMI. Pediatr Obes 2018;13:598-606.PMC6390491
110. Much D, Brunner S, Vollhardt C, et al. Breast milk fatty acid profile in relation to infant growth and body composition: results from the INFAT study. Pediatr Res 2013;74:230-7
111. Robinson DT, Palac HL, Baillif V, et al. Long chain fatty acids and related pro-inflammatory, specialized pro-resolving lipid mediators and their intermediates in preterm human milk during the first month of lactation. Prostaglandins Leukot Essent Fatty Acids 2017;121:1-6
112. Badiou S, Tuaillon E, Viljoen J, et al. Association between breast milk fatty acids and HIV-1 transmission through breastfeeding. Prostaglandins Leukot Essent Fatty Acids 2016;105:35-42
113. Villamor E, Koulinska IN, Furtado J, et al. Long-chain n-6 polyunsaturated fatty acids in breast milk decrease the risk of HIV transmission through breastfeeding. Am J Clin Nutr 2007;86:682-9
114. Miliku K, Duan QL, Moraes TJ, et al. Human milk fatty acid composition is associated with dietary, genetic, sociodemographic, and environmental factors in the CHILD Cohort Study. Am J Clin Nutr 2019;110:1370-83.PMC6885479
115. Guidelines on HIV and Infant Feeding 2010: Principles and Recommendations for Infant Feeding in the Context of HIV and a Summary of Evidence. Geneva2010.
116. Prentice RL. A case-cohort design for epidemiologic cohort studies and disease prevention Biometrika 1986;73:1-11
117. Kupper LL MA, Spirtas R. A Hybrid Epidemiologic Study Design Useful in Estimating Relative Risk. Journal of the American Statistical Association 1975;70:524-8
118. Langholz B, Thomas DC. Nested case-control and case-cohort methods of sampling from a cohort: a critical comparison. Am J Epidemiol 1990;131:169-76
119. Wacholder S. Practical considerations in choosing between the case-cohort and nested case-control designs. Epidemiology 1991;2:155-8
120. Rundle AG, Vineis P, Ahsan H. Design options for molecular epidemiology research within cohort studies. Cancer Epidemiol Biomarkers Prev 2005;14:1899-907
121. Colbers A, Mirochnick M, Schalkwijk S, Penazzato M, Townsend C, Burger D. Importance of Prospective Studies in Pregnant and Breastfeeding Women Living With Human Immunodeficiency Virus. Clin Infect Dis 2019;69:1254-8.PMC6743813
122. Krubiner CB, Faden RR, Cadigan RJ, et al. Advancing HIV research with pregnant women: navigating challenges and opportunities. AIDS 2016;30:2261-5.PMC5014683
123. Westreich D, Rosenberg M, Schwartz S, Swamy G. Representation of women and pregnant women in HIV research: a limited systematic review. PLoS One 2013;8:e73398.PMC3751870
124. Gupta A, Mathad JS, Abdel-Rahman SM, et al. Toward Earlier Inclusion of Pregnant and Postpartum Women in Tuberculosis Drug Trials: Consensus Statements From an International Expert Panel. Clin Infect Dis 2016;62:761-9.PMC4772846
125. Sharma V, Sharma P, Sharma S. Managing bipolar disorder during pregnancy and the postpartum period: a critical review of current practice. Expert Rev Neurother 2020;20:373-83
126. Santarsieri D, Schwartz TL. Antidepressant efficacy and side-effect burden: a quick guide for clinicians. Drugs Context 2015;4:212290.PMC4630974
127. Uguz F. Antipsychotic Use During Pregnancy and the Risk of Gestational Diabetes Mellitus: A Systematic Review. J Clin Psychopharmacol 2019;39:162-7
128. Kulkarni J, Storch A, Baraniuk A, Gilbert H, Gavrilidis E, Worsley R. Antipsychotic use in pregnancy. Expert Opin Pharmacother 2015;16:1335-45
129. Musil R, Obermeier M, Russ P, Hamerle M. Weight gain and antipsychotics: a drug safety review. Expert Opin Drug Saf 2015;14:73-96
130. Grootens KP, Meijer A, Hartong EG, et al. Weight changes associated with antiepileptic mood stabilizers in the treatment of bipolar disorder. Eur J Clin Pharmacol 2018;74:1485-9
131. Kolstad E, Veiby G, Gilhus NE, Bjork M. Overweight in epilepsy as a risk factor for pregnancy and delivery complications. Epilepsia 2016;57:1849-57
132. Jao J, Abrams EJ, Phillips T, Petro G, Zerbe A, Myer L. In Utero Tenofovir Exposure Is not Associated With Fetal Long Bone Growth. Clin Infect Dis 2016;62:1604-9.PMC4885649
133. Le Roux SM, Jao J, Brittain K, et al. Tenofovir exposure in utero and linear growth in HIV-exposed, uninfected infants. AIDS 2017;31:97-104.PMC5814299
134. Jao J, Powis KM, Kirmse B, et al. Lower mitochondrial DNA and altered mitochondrial fuel metabolism in HIV-exposed uninfected infants in Cameroon. AIDS 2017;31:2475-81.5680102
135. Bengtson AM, Phillips TK, le Roux SM, et al. Postpartum obesity and weight gain among human immunodeficiency virus-infected and human immunodeficiency virus-uninfected women in South Africa. Matern Child Nutr 2020:e12949
136. Myer L, Dunning L, Lesosky M, et al. Frequency of Viremic Episodes in HIV-Infected Women Initiating Antiretroviral Therapy During Pregnancy: A Cohort Study. Clin Infect Dis 2017;64:422-7.PMC5849096
137. Mahtab S, Zar HJ, Ntusi NAB, et al. Endothelial dysfunction in South African youth living with perinatally acquired HIV on antiretroviral therapy. Clin Infect Dis 2020
138. Frigati LJ, Jao J, Mahtab S, et al. Insulin Resistance in South African Youth Living with Perinatally Acquired HIV Receiving Antiretroviral Therapy. AIDS Res Hum Retroviruses 2019;35:56-62.PMC6343194
139. Siri WE. Body composition from fluid spaces and density: analysis of methods. 1961. Nutrition 1993;9:480-91; discussion , 92

140. de Castro JAC, de Lima LRA, Silva DAS. Accuracy of octa-polar bioelectrical impedance analysis for the assessment of total and appendicular body composition in children and adolescents with HIV: comparison with dual energy X-ray absorptiometry and air displacement plethysmography. J Hum Nutr Diet 2018;31:276-85
141. PrayGod G, Changalucha J, Kapiga S, Peck R, Todd J, Filteau S. Dysglycemia associations with adipose tissue among HIV-infected patients after 2 years of antiretroviral therapy in Mwanza: a follow-up cross-sectional study. BMC infectious diseases 2017;17:103.PMC5282875
142. Markos Y GT, Shegaze, M. Validity of Prediction of Thoracic Gas Volume and Body Composition Using Air Displacement Plethysmography in People Living with HIV in Southwest Ethiopia. Pulmonary and Respiratory Medicine 2017;7:2
143. Castillo H, Santos IS, Matijasevich A. Relationship between maternal pre-pregnancy body mass index, gestational weight gain and childhood fatness at 6-7 years by air displacement plethysmography. Matern Child Nutr 2015;11:606-17.PMC4832361
144. Catalano PM, Wong WW, Drago NM, Amini SB. Estimating body composition in late gestation: a new hydration constant for body density and total body water. Am J Physiol 1995;268:E153-8
145. Forsum E, Henriksson P, Lof M. The two-component model for calculating total body fat from body density: an evaluation in healthy women before, during and after pregnancy. Nutrients 2014;6:5888-99.PMC4277005
146. Henriksson P, Lof M, Forsum E. Assessment and prediction of thoracic gas volume in pregnant women: an evaluation in relation to body composition assessment using air displacement plethysmography. Br J Nutr 2013;109:111-7
147. Norris T, Ramel SE, Catalano P, et al. New charts for the assessment of body composition, according to air-displacement plethysmography, at birth and across the first 6 mo of life. Am J Clin Nutr 2019;109:1353-60
148. Josefson JL, Nodzenski M, Talbot O, Scholtens DM, Catalano P. Fat mass estimation in neonates: anthropometric models compared with air displacement plethysmography. Br J Nutr 2019;121:285-90.PMC7009914
149. Schneider CR, Catalano PM, Biggio JR, Gower BA, Chandler-Laney PC. Associations of neonatal adiponectin and leptin with growth and body composition in African American infants. Pediatr Obes 2018;13:485-91.PMC6457440
150. Catalano PM, Thomas AJ, Avallone DA, Amini SB. Anthropometric estimation of neonatal body composition. Am J Obstet Gynecol 1995;173:1176-81
151. Huston Presley L, Wong WW, Roman NM, Amini SB, Catalano PM. Anthropometric estimation of maternal body composition in late gestation. Obstet Gynecol 2000;96:33-7
152. Ehrenberg HM, Huston-Presley L, Catalano PM. The influence of obesity and gestational diabetes mellitus on accretion and the distribution of adipose tissue in pregnancy. Am J Obstet Gynecol 2003;189:944-8
153. Lindsay CA, Huston L, Amini SB, Catalano PM. Longitudinal changes in the relationship between body mass index and percent body fat in pregnancy. Obstet Gynecol 1997;89:377-82
154. Madlala HP, Malaba TR, Newell ML, Myer L. Elevated body mass index during pregnancy and gestational weight gain in HIV-infected and HIV-uninfected women in Cape Town, South Africa: association with adverse birth outcomes. Trop Med Int Health 2020
155. Mari A, Pacini G, Murphy E, Ludvik B, Nolan JJ. A model-based method for assessing insulin sensitivity from the oral glucose tolerance test. Diabetes Care 2001;24:539-48
156. Mari A, Ferrannini E. Beta-cell function assessment from modelling of oral tests: an effective approach. Diabetes Obes Metab 2008;10 Suppl 4:77-87
157. Tura A, Mari A, Prikoszovich T, Pacini G, Kautzky-Willer A. Value of the intravenous and oral glucose tolerance tests for detecting subtle impairments in insulin sensitivity and beta-cell function in former gestational diabetes. Clin Endocrinol (Oxf) 2008;69:237-43
158. Matsuda M, DeFronzo RA. Insulin sensitivity indices obtained from oral glucose tolerance testing: comparison with the euglycemic insulin clamp. Diabetes Care 1999;22:1462-70
159. Kirwan JP, Huston-Presley L, Kalhan SC, Catalano PM. Clinically useful estimates of insulin sensitivity during pregnancy: validation studies in women with normal glucose tolerance and gestational diabetes mellitus. Diabetes Care 2001;24:1602-7
160. Utzschneider KM, Prigeon RL, Faulenbach MV, et al. Oral disposition index predicts the development of future diabetes above and beyond fasting and 2-h glucose levels. Diabetes Care 2009;32:335-41.PMC2628704
161. American Diabetes Association. Standards of Medical Care in Diabetes 2017. Diabetes Care 2017;40, Supplement 1:S1-S135
162. International Association of D, Pregnancy Study Groups Consensus P, Metzger BE, et al. International association of diabetes and pregnancy study groups recommendations on the diagnosis and classification of hyperglycemia in pregnancy. Diabetes Care 2010;33:676-82.PMC2827530
163. Matthews DR, Hosker JP, Rudenski AS, Naylor BA, Treacher DF, Turner RC. Homeostasis model assessment: insulin resistance and beta-cell function from fasting plasma glucose and insulin concentrations in man. Diabetologia 1985;28:412-9
164. Grundy SM, Stone NJ, Bailey AL, et al. 2018 AHA/ACC/AACVPR/AAPA/ABC/ACPM/ADA/AGS/APhA/ASPC/NLA/PCNA Guideline on the Management of Blood Cholesterol: A Report of the American College of Cardiology/American Heart Association Task Force on Clinical Practice Guidelines. Circulation 2019;139:e1082-e143
165. Myer L, Stein DJ, Grimsrud A, Seedat S, Williams DR. Social determinants of psychological distress in a nationally-representative sample of South African adults. Social science & medicine 2008;66:1828-40.PMC3203636
166. Brittain K, Remien RH, Phillips T, et al. Factors associated with alcohol use prior to and during pregnancy among HIV-infected pregnant women in Cape Town, South Africa. Drug Alcohol Depend 2017;173:69-77.PMC5429399
167. Kalk E, Heekes A, Mehta U, et al. Safety and Effectiveness of Isoniazid Preventive Therapy in HIV-Positive Pregnant Women on Art: An Observational Study using Linked Population Data. Clin Infect Dis 2020
168. Data centre profile: the Provincial Health Data Centre of the Western Cape Province, South Africa. International Journal of Population Data Science 2019;4 (Special Issue: Data Centre Profiles)
169. Heekes A, Tiffin N, Dane P, et al. Self-enrolment antenatal health promotion data as an adjunct to maternal clinical information systems in the Western Cape Province of South Africa. BMJ Glob Health 2018;3:e000565.PMC5922480
170. Odayar J, Rangaka MX, Zerbe A, et al. Burden of tuberculosis in HIV-positive pregnant women in Cape Town, South Africa. Int J Tuberc Lung Dis 2018;22:760-5
171. Lesosky M, Glass T, Rambau B, Hsiao NY, Abrams EJ, Myer L. Bias in the estimation of cumulative viremia in cohort studies of HIV-infected individuals. Ann Epidemiol 2019;38:22-7.PMC6812622
172. Esser N, Legrand-Poels S, Piette J, Scheen AJ, Paquot N. Inflammation as a link between obesity, metabolic syndrome and type 2 diabetes. Diabetes Res Clin Pract 2014;105:141-50

173. Ostrowski SR, Katzenstein TL, Pedersen BK, Gerstoft J, Ullum H. Residual viraemia in HIV-1-infected patients with plasma viral load <or=20 copies/ml is associated with increased blood levels of soluble immune activation markers. Scand J Immunol 2008;68:652-60
174. Pradhan AD, Manson JE, Rifai N, Buring JE, Ridker PM. C-reactive protein, interleukin 6, and risk of developing type 2 diabetes mellitus. JAMA 2001;286:327-34
175. Phillips TK, Sinxadi P, Abrams EJ, et al. A Comparison of Plasma Efavirenz and Tenofovir, Dried Blood Spot Tenofovir-Diphosphate, and Self-Reported Adherence to Predict Virologic Suppression Among South African Women. J Acquir Immune Defic Syndr 2019;81:311-8.PMC6565450
176. Phillips TK, Wilson IB, Brittain K, et al. Decreases in Self-Reported ART Adherence Predict HIV Viremia Among Pregnant and Postpartum South African Women. J Acquir Immune Defic Syndr 2019;80:247-54.PMC6375758
177. Phillips T, Brittain K, Mellins CA, et al. A Self-Reported Adherence Measure to Screen for Elevated HIV Viral Load in Pregnant and Postpartum Women on Antiretroviral Therapy. AIDS and behavior 2017;21:450-61.PMC5145763
178. Phillips T RA, Reynolds SJ, Hsiao NYM, Maartens G, Abrams EJ, Myer L. Routine pharmacy refills predict women's plasma ARV detection and viral suppression. Poster #1083. Presented at the Conference on Retroviruses and Opportunistic Infections, March, 2020, Boston, MA, USA.
179. le Roux SM, Abrams EJ, Nguyen KK, Myer L. HIV incidence during breastfeeding and mother-to-child transmission in Cape Town, South Africa. AIDS 2019;33:1399-401
180. Zash R, Holmes L, Diseko M, et al. Neural-Tube Defects and Antiretroviral Treatment Regimens in Botswana. N Engl J Med 2019;381:827-40.PMC6995896
181. Villar J, Cheikh Ismail L, Victora CG, et al. International standards for newborn weight, length, and head circumference by gestational age and sex: the Newborn Cross-Sectional Study of the INTERGROWTH-21st Project. Lancet 2014;384:857-68
182. Hong YH, Chung S. Small for gestational age and obesity related comorbidities. Ann Pediatr Endocrinol Metab 2018;23:4-8.PMC5894558
183. Markopoulou P, Papanikolaou E, Analytis A, Zoumakis E, Siahanidou T. Preterm Birth as a Risk Factor for Metabolic Syndrome and Cardiovascular Disease in Adult Life: A Systematic Review and Meta-Analysis. The Journal of pediatrics 2019;210:69-80 e5
184. le Roux SM, Abrams EJ, Donald KA, et al. Infectious morbidity of breastfed, HIV-exposed uninfected infants under conditions of universal antiretroviral therapy in South Africa: a prospective cohort study. Lancet Child Adolesc Health 2020;4:220-31.PMC7235356
185. https://informprojectsite.github.io/INFORM. Last accessed April 20, 2020.
186. Grivell, RM, Yelland LN, Deussen A, et al. Antenatal dietary and lifestyle advice for women who are overweight or obese and the effect on fetal growth and adiposity: the LIMIT randomised trial. Int J Obstet Gynaecol 2016;123(2):233-243.
187. Ferraioli G, Soares Monteiro LB. Ultrasound-based techniques for the diagnosis of liver steatosis. World journal of gastroenterology 2019;25:6053-62.PMC6824276
188. Joseph AE, Saverymuttu SH, al-Sam S, Cook MG, Maxwell JD. Comparison of liver histology with ultrasonography in assessing diffuse parenchymal liver disease. Clin Radiol 1991;43:26-31
189. Santini F, Giannetti M, Mazzeo S, et al. Ultrasonographic evaluation of liver volume and the metabolic syndrome in obese women. J Endocrinol Invest 2007;30:104-10
190. Mogoba P, Gomba Y, Brittain K, et al. Re-recruiting postpartum women living with HIV into a follow-up study in Cape Town, South Africa. BMC Res Notes 2019;12:461.PMC6660934
191. le Roux SM, Abrams EJ, Donald KA, et al. Growth trajectories of breastfed HIV-exposed uninfected and HIV-unexposed children under conditions of universal maternal antiretroviral therapy: a prospective study. Lancet Child Adolesc Health 2019;3:234-44
192. Myer L, Phillips TK, Zerbe A, et al. Integration of postpartum healthcare services for HIV-infected women and their infants in South Africa: A randomised controlled trial. PLoS medicine 2018;15:e1002547.PMC5877834
193. Odayar J, Myer L, Malaba TR, Kabanda S, Allerton J, Hu NC, Mukonda E, Fourie S, Jacobs S, Huang D, Kalombo C, Hsiao NY, Myer L, for the Postpartum Adherence Clubs for Antiretroviral Therapy (PACART) Trial. Differentiated care for postpartum ART in South African women living with HIV: an RCT. Paper 131LB presented at the Conference on Retroviruses and Opportunistic Infections (CROI), March 2020, Boston, MA, USA.
194. Malaba TR, Gray CM, Myer L, Newell M-L. Cohort Profile: Prematurity Immunology in HIV-infected Mothers and their infants Study (PIMS). 2020:2020.03.18.20033654
195. Vittinghoff E, Sen S, McCulloch CE. Sample size calculations for evaluating mediation. Stat Med 2009;28:541-57
196. Perkins NJ, Cole SR, Harel O, et al. Principled Approaches to Missing Data in Epidemiologic Studies. Am J Epidemiol 2018;187:568-75.PMC5860376
197. Sankoh AJ, D'Agostino RB, Sr., Huque MF. Efficacy endpoint selection and multiplicity adjustment methods in clinical trials with inherent multiple endpoint issues. Stat Med 2003;22:3133-50
198. Sankoh AJ, Huque MF, Dubey SD. Some comments on frequently used multiple endpoint adjustment methods in clinical trials. Stat Med 1997;16:2529-42
199. Hommel G. A Stagewise Rejective Multiple Test Procedure Based on a Modified Bonferroni Test. Accessed at http://www.jstor.org/stable/2336190 on May 8, 2020. Biometrika 1988;75:383-6
200. Benjamini Y HY. Controlling the False Discovery Rate - a Practical and Powerful Approach to Multiple Testing. J R Stat Soc Ser B-Methodol 1995;57:289-300
201. Hochberg Y, Benjamini Y. More powerful procedures for multiple significance testing. Stat Med 1990;9:811-8
202. Shaffer ML, Kunselman AR, Watterberg KL. Analysis of neonatal clinical trials with twin births. BMC Med Res Methodol 2009;9:12.PMC2676314
203. Breslow NE and Clayton DG. Approximate Inference in Generalized Linear Mixed Models. Journal of the American Statistical Association, vol. 88, no. 421, 1993, pp. 9–25. JSTOR, www.jstor.org/stable/2290687. Accessed 25 May 2020.
204. VanderWeele TJ, Hernan MA, Robins JM. Causal directed acyclic graphs and the direction of unmeasured confounding bias. Epidemiology 2008;19:720-8.PMC4242711
205. Schisterman EF, Cole SR, Platt RW. Overadjustment bias and unnecessary adjustment in epidemiologic studies. Epidemiology 2009;20:488-95.PMC2744485
206. Howards PP, Schisterman EF, Poole C, Kaufman JS, Weinberg CR. "Toward a clearer definition of confounding" revisited with directed acyclic graphs. Am J Epidemiol 2012;176:506-11.PMC3530354
207. Lee K, Small DS, Rosenbaum PR. A powerful approach to the study of moderate effect modification in observational studies. Biometrics 2018;74:1161-70
208. IOM (Institute of Medicine) and NRC (National Research Council). Weight Gain During Pregnancy: Reexamining the Guidelines. Washington DC: National Academies Press; 2009.

209. Richiardi L, Bellocco R, Zugna D. Mediation analysis in epidemiology: methods, interpretation and bias. Int J Epidemiol 2013;42:1511-9
210. Vanderweele TJ, Vansteelandt S, Robins JM. Effect decomposition in the presence of an exposure-induced mediator-outcome confounder. Epidemiology 2014;25:300-6.PMC4214081
211. Derkach A, Moore SC, Boca SM, Sampson JN. Group testing in mediation analysis. Stat Med 2020
212. Imai K, Keele L, Tingley D. A general approach to causal mediation analysis. Psychol Methods 2010;15:309-34
213. Gaynor SM, Schwartz J, Lin X. Mediation analysis for common binary outcomes. Stat Med 2019;38:512-29
214. Tchetgen Tchetgen EJ, Vanderweele TJ. Identification of natural direct effects when a confounder of the mediator is directly affected by exposure. Epidemiology 2014;25:282-91.PMC4230499
215. Kim C, Daniels M, Li Y, Milbury K, Cohen L. A Bayesian semiparametric latent variable approach to causal mediation. Stat Med 2018;37:1149-61.PMC5837944
216. Lefebvre G, Samoilenko M, Boucoiran I, Blais L. A Bayesian finite mixture of bivariate regression model for causal mediation analyses. Stat Med 2018;37:3637-60
217. Lindmark A, de Luna X, Eriksson M. Sensitivity analysis for unobserved confounding of direct and indirect effects using uncertainty intervals. Stat Med 2018;37:1744-62
218. Indirect calorimetry as point of care testing. Singer P, Rattanachaiwong S. Clinical Nutrition. 2019
219. The clinical evaluation of the new indirect calorimeter developed by the ICALIC project. Oshima T, et al. Clin Nutr 2020
220. Steyn NP, Jaffer N, Nel J, et al. Dietary Intake of the Urban Black Population of Cape Town: The Cardiovascular Risk in Black South Africans (CRIBSA) Study. Nutrients 2016;8.PMC4882698
221. www.safoods.mrc.ac.za Last accessed April 20, 2020.
222. Chen K, Lo S-H. Case-cohort and case-control analysis with Cox's model. Biometrika 1999;86:755-64
223. Chen K. Generalized case–cohort sampling. 2001;63:791-809
224. Barlow WE, Ichikawa L, Rosner D, Izumi S. Analysis of case-cohort designs. J Clin Epidemiol 1999;52:1165-72
225. Barlow WE. Robust variance estimation for the case-cohort design. Biometrics 1994;50:1064-72
226. Breslow NE, Lumley T, Ballantyne CM, Chambless LE, Kulich M. Using the whole cohort in the analysis of case-cohort data. Am J Epidemiol 2009;169:1398-405.PMC2768499
227. Daniel RM, De Stavola BL, Cousens SN, Vansteelandt S. Causal mediation analysis with multiple mediators. Biometrics 2015;71:1-14.PMC4402024
228. Cole SR, Hudgens MG, Tien PC, et al. Marginal structural models for case-cohort study designs to estimate the association of antiretroviral therapy initiation with incident AIDS or death. Am J Epidemiol 2012;175:381-90.PMC3282878
229. Shikuma CM, Gangcuangco LM, Killebrew DA, et al. The role of HIV and monocytes/macrophages in adipose tissue biology. J Acquir Immune Defic Syndr 2014;65:151-9.PMC4020346
230. Gojanovich GS, Shikuma CM, Milne C, Libutti DE, Chow DC, Gerschenson M. Subcutaneous Adipocyte Adenosine Triphosphate Levels in HIV Infected Patients. AIDS Res Hum Retroviruses 2020;36:75-82.PMC6944137
231. Basu S, Haghiac M, Surace P, et al. Pregravid obesity associates with increased maternal endotoxemia and metabolic inflammation. Obesity (Silver Spring) 2011;19:476-82.PMC3628602
232. Resi V, Basu S, Haghiac M, et al. Molecular inflammation and adipose tissue matrix remodeling precede physiological adaptations to pregnancy. American journal of physiology Endocrinology and metabolism 2012;303:E832-40.PMC3469618
233. Radikova Z, Koska J, Huckova M, et al. Insulin sensitivity indices: a proposal of cut-off points for simple identification of insulin-resistant subjects. Experimental and clinical endocrinology & diabetes : official journal, German Society of Endocrinology [and] German Diabetes Association 2006;114:249-56
234. Enot DP, Haas B, Weinberger KM. Bioinformatics for mass spectrometry-based metabolomics. Methods Mol Biol 2011;719:351-75
235. Kelly RS, McGeachie MJ, Lee-Sarwar KA, et al. Partial Least Squares Discriminant Analysis and Bayesian Networks for Metabolomic Prediction of Childhood Asthma. Metabolites 2018;8.PMC6316795
236. Rosato A, Tenori L, Cascante M, De Atauri Carulla PR, Martins Dos Santos VAP, Saccenti E. From correlation to causation: analysis of metabolomics data using systems biology approaches. Metabolomics 2018;14:37.PMC5829120
237. Gerl MJ, Klose C, Surma MA, et al. Machine learning of human plasma lipidomes for obesity estimation in a large population cohort. PLoS Biol 2019;17:e3000443.PMC6799887 following competing interests: KS is CEO of Lipotype GmbH. KS, CK and MS are shareholders of Lipotype GmbH. MJG is employee of Lipotype GmbH. VS has participated in a conference trip sponsored by Novo Nordisk and received an honorarium from the same source for participating in an advisory board meeting. He also has ongoing research collaboration with Bayer Ltd.
238. Amato U, Antoniadis A, De Feis I, Gijbels I. Penalised robust estimators for sparse and high-dimensional linear models. Statistical Methods & Applications 2020
239. Yu G, Yin L, Lu S, Liu Y. Confidence Intervals for Sparse Penalized Regression With Random Designs. Journal of the American Statistical Association 2019:1-38
240. Yu Y, Feng Y. Modified Cross-Validation for Penalized High-Dimensional Linear Regression Models. Journal of Computational and Graphical Statistics 2014;23:1009-27
241. Jung Y, Huang JZ, Hu J. Biomarker Detection in Association Studies: Modeling SNPs Simultaneously via Logistic ANOVA. J Am Stat Assoc 2014;109:1355-67.PMC4310485
242. Jung Y, Zhang H, Hu J. Transformed low-rank ANOVA models for high-dimensional variable selection. Stat Methods Med Res 2019;28:1230-46
243. Castelletti F, Consonni G. Bayesian inference of causal effects from observational data in Gaussian graphical models. Biometrics 2020
244. Liu J, Yu G, Liu Y. Graph-based sparse linear discriminant analysis for high-dimensional classification. J Multivar Anal 2019;171:250-69.PMC6980367
245. Langfelder P, Horvath S. WGCNA: an R package for weighted correlation network analysis. BMC Bioinformatics 2008;9:559.PMC2631488
246. Tremblay BL, Guenard F, Lamarche B, Perusse L, Vohl MC. Weighted gene co-expression network analysis to explain the relationship between plasma total carotenoids and lipid profile. Genes Nutr 2019;14:16.PMC6505263
247. Langfelder P, Zhang B, Horvath S. Defining clusters from a hierarchical cluster tree: the Dynamic Tree Cut package for R. Bioinformatics 2008;24:719-20
248. Song Y, Zhou X, Zhang M, et al. Bayesian shrinkage estimation of high dimensional causal mediation effects in omics studies. Biometrics 2019
249. Derkach A, Pfeiffer RM, Chen TH, Sampson JN. High dimensional mediation analysis with latent variables. Biometrics 2019;75:745-56

250. Luo C, Fa B, Yan Y, et al. High-dimensional mediation analysis in survival models. PLoS Comput Biol 2020;16:e1007768.PMC7190184
251. Penchala SD, Fawcett S, Else L, et al. The development and application of a novel LC-MS/MS method for the measurement of Dolutegravir, Elvitegravir and Cobicistat in human plasma. Journal of chromatography B, Analytical technologies in the biomedical and life sciences 2016;1027:174-80
252. NICHD Strategic Plan 2020. Healthy pregnancies. Healthy children. Healthy optimal lives. accessed at https://www.nichd.nih.gov/about/org/strategicplan on Dec 18, 2019. .
253. Myer L, Redd AD, Mukonda E, et al. Antiretroviral Adherence, Elevated Viral Load, and Drug Resistance Mutations in Human Immunodeficiency Virus-infected Women Initiating Treatment in Pregnancy: A Nested Case-control Study. Clin Infect Dis 2020;70:501-8.PMC7188229
254. Geffner ME, Patel K, Jacobson DL, et al. Changes in Insulin Sensitivity over Time and Associated Factors in HIV-Infected Adolescents. AIDS 2017
255. Lowe WL, Jr., Lowe LP, Kuang A, et al. Maternal glucose levels during pregnancy and childhood adiposity in the Hyperglycemia and Adverse Pregnancy Outcome Follow-up Study. Diabetologia 2019;62:598-610.PMC6421132
256. McComsey GA, Daar ES, O'Riordan M, et al. Changes in fat mitochondrial DNA and function in subjects randomized to abacavir-lamivudine or tenofovir DF-emtricitabine with atazanavir-ritonavir or efavirenz: AIDS Clinical Trials Group study A5224s, substudy of A5202. J Infect Dis 2013;207:604-11.PMC3549598
257. Robinson DT, Josefson J, Van Horn L. Considerations for Preterm Human Milk Feedings When Caring for Mothers Who Are Overweight or Obese. Adv Neonatal Care 2019;19:361-70
